# Severe Outcomes Associated With SARS-CoV-2 Infection in Children: A Systematic Review and Meta-Analysis

**DOI:** 10.3389/fped.2022.916655

**Published:** 2022-06-09

**Authors:** Madeleine W. Sumner, Alicia Kanngiesser, Kosar Lotfali-Khani, Nidhi Lodha, Diane Lorenzetti, Anna L. Funk, Stephen B. Freedman

**Affiliations:** ^1^Schulich School of Medicine and Dentistry, Western University, London, ON, Canada; ^2^Section of Pediatric Emergency Medicine, Department of Pediatrics, Cumming School of Medicine, University of Calgary, Calgary, AB, Canada; ^3^Department of Biological Sciences, University of Calgary, Calgary, AB, Canada; ^4^Health Sciences Library and Department of Community Health Sciences, Cumming School of Medicine, University of Calgary, Calgary, AB, Canada; ^5^Sections of Pediatric Emergency Medicine and Gastroenterology, Departments of Pediatrics and Emergency Medicine, Cumming School of Medicine, University of Calgary, Calgary, AB, Canada

**Keywords:** COVID-19, meta-analysis, SARS-CoV-2, severity, outcomes, children

## Abstract

**Objective:**

To estimate the proportion of SARS-CoV-2 infected children experiencing hospitalization, intensive care unit (ICU) admission, severe outcomes, and death.

**Data Sources:**

PubMed, Embase, and MedRxiv were searched for studies published between December 1, 2019 and May 28, 2021. References of relevant systematic reviews were also screened.

**Study Selection:**

We included cohort or cross-sectional studies reporting on at least one outcome measure (i.e., hospitalization, ICU admission, severe outcomes, death) for ≥100 children ≤21 years old within 28 days of SARS-CoV-2 positivity; no language restrictions were applied.

**Data Extraction and Synthesis:**

Two independent reviewers performed data extraction and risk of bias assessment. Estimates were pooled using random effects models. We adhered to the Preferred Reporting Items for Systematic Reviews and Meta-Analysis (PRISMA) guidelines.

**Main Outcomes and Measures:**

Percentage of SARS-CoV-2 positive children experiencing hospitalization, ICU admission, severe outcome, and death.

**Results:**

118 studies representing 3,324,851 SARS-CoV-2 infected children from 68 countries were included. Community-based studies (*N* = 48) reported that 3.3% (95%CI: 2.7–4.0%) of children were hospitalized, 0.3% (95%CI: 0.2–0.6%) were admitted to the ICU, 0.1% (95%CI: 0.0–2.2%) experienced a “severe” outcome and 0.02% (95%CI: 0.001–0.05%) died. Hospital-based screening studies (*N* = 39) reported that 23.9% (95%CI: 19.0–29.2%) of children were hospitalized, 2.9% (95%CI: 2.1–3.8%) were admitted to the ICU, 1.3% (95%CI: 0.5–2.3%) experienced a severe outcome, and 0.2% (95%CI: 0.02–0.5%) died. Studies of hospitalized children (*N* = 31) reported that 10.1% (95%CI: 6.1–14.9%) of children required ICU admission, 4.2% (95%CI: 0.0–13.8%) had a severe outcome and 1.1% (95%CI: 0.2–2.3%) died. Low risk of bias studies, those from high-income countries, and those reporting outcomes later in the pandemic presented lower estimates. However, studies reporting outcomes after May 31, 2020, compared to earlier publications, had higher proportions of hospitalized patients requiring ICU admission and experiencing severe outcomes.

**Conclusion and Relevance:**

Among children tested positive for SARS-CoV-2, 3.3% were hospitalized, with rates being higher early in the pandemic. Severe outcomes, ICU admission and death were uncommon, however estimates vary by study population, pandemic timing, study risk of bias, and economic status of the country.

**Systematic Review Registration:**

PROSPERO, identifier [CRD42021260164].

## Introduction

As the COVID pandemic has progressed, children have represented an expanding population of those infected, with those <20 years old representing 20% of reported cases worldwide, and 0.4% of deaths (1). In the United States, this proportion is approximately 19.0% (2). Although the clinical course of SARS-CoV-2 infection in children is generally mild, hospitalization, intensive care unit (ICU) admission, and death, do occur (3–7). Several studies have examined severe outcomes among children with COVID-19; however, estimates vary widely based on testing strategies, study populations, and outcome definitions. While 2021 Centers for Disease Control and Prevention (CDC) data estimates that 2.3% of children <18 years old in the United States infected with SARS-CoV-2 are hospitalized, with 0.8% requiring intensive care, and <0.1% dying (3), the Public Health Agency of Canada data reports lower rates (0.5, 0.07, and 0.005%, respectively) (8). Moreover, meta-analyses estimate higher rates of severe outcomes than the CDC (9–11). Higher death and ICU rates have also been reported in low-/middle-income countries (LMIC) compared to high income countries (12).

To provide clarity regarding the risk of severe outcomes in children, we performed a systematic review and meta-analysis focused on estimating the risk of severe or critical outcomes among children infected with SARS-CoV-2 and provide study setting-based [i.e., population-level, hospital, and inpatient] pooled risk estimates. Secondary objectives were to explore sources of between-study heterogeneity through subgroup analyses stratified by risk of bias (RoB), country economic status, and data collection time-period.

## Methods

The protocol for this review was registered with PROSPERO (CRD42021260164) and this report adhered to the Preferred Reporting Items of Systematic Review and Meta-analysis guidelines (13).

### Search Strategy

Electronic searches of PubMed, EMBASE (*via* Ovid), and MedRxiv for studies published between December 1, 2019 and May 28, 2021, were conducted with the assistance of a medical librarian (DL). Our search terms aimed to identify studies reporting on clinical outcomes of SARS-CoV-2 infection or COVID-19 in children, without restriction by subject area (see Supplementary Table 1 for search strategy). We performed a manual search of the references of systematic reviews describing relevant aspects of SARS-CoV-2 infection in children to identify additional eligible studies. There were no language or geographic restrictions. Given the large number of publications identified, we did not search for unpublished studies or contact experts in the field.

### Eligibility Criteria

Eligible studies reported on the proportions of children experiencing severe COVID-19 outcomes. Eligibility criteria were: (1) ≥100 children and young adults ≤21 years of age with positive SARS-CoV-2 nucleic acid and/or antigen tests; and (2) reported ≥1 outcome of interest (i.e., hospitalization, ICU admission, severe outcome per study definition, and death) occurring within 28 days of SARS-CoV-2 positivity. Observational studies, including prospective and retrospective cohort and cross-sectional studies were eligible. Case-control, case series, case report studies, and modeling studies with no original data were excluded. Studies reported in a language other than English were translated and examined for eligibility by two independent reviewers (MS, AK, KL-K, or NL).

### Outcomes

We targeted four measures of disease severity - hospitalization, ICU admission, severe outcome, and death. Sub-group analyses were performed based on study RoB, economic status of study country, and timing of the pandemic.

### Data Extraction

Articles underwent title/abstract screening, full text review, and data extraction in duplicate by two of four randomly selected independent reviewers (MS, KL-K, NL, or AK). Agreement was required for studies to advance to full text review and similarly to data extraction. Disagreements and discrepancies were discussed at weekly team meetings until consensus was achieved. Study screening, data extraction and RoB assessment data were entered into a Covidence 2.0 database.

Studies that did not explicitly meet eligibility criteria based on the published paper but included data that could contribute to our analysis (e.g., mixed pediatric-adult studies without age-disaggregated data) were flagged. The corresponding authors of these studies were contacted twice by e-mail, 2-weeks apart, to inquire if they could provide the data required. If there was no reply after 28 days, the paper was excluded.

### Datasets and Overlapping Populations

Given the wide availability of COVID-19 datasets, many studies reported data from the same source. To minimize the inclusion of duplicate data, we carefully examined all non-original data to identify the original data source. When duplicate data sets were identified, we included the study with the greatest number of children that reported on at least one of our outcome measures. We excluded studies reporting on data from regional ministries of health if data from a national governmental source were included from another study (e.g., New York Department of Health data that were likely included in CDC data). We did include data from individual hospitals, hospital networks and insurance databases as these sources were deemed unlikely to directly overlap with national surveillance sources.

### Definitions

Population-based studies were defined as those based on public health reporting systems (national or regional) or those recruiting children outside of hospital settings. We designated studies as “hospital-based screening” if participants were recruited at a hospital and both outpatients and inpatient outcomes were recorded (e.g., emergency departments). Studies reporting on solely hospital inpatients were analyzed separately.

Severe outcome was defined by reviewing each study’s own definition of “severe” or “critical” disease and looking for commonalities. We included studies in the severe outcome analysis if their definition aligned with the following cluster of symptoms: respiratory failure, acute respiratory distress syndrome (ARDS), coma or encephalopathy, shock, myocardial injury, heart failure, coagulation dysfunction, acute kidney injury (AKI), and/or life-threatening multiorgan dysfunction requiring interventions such as invasive mechanical ventilation (IMV) or extracorporeal membrane oxygenation (ECMO). The studies included in the “severe” outcome analysis and their definitions are provided (Table 1).

**TABLE 1:** Definition of severe outcome in the analyzed studies.

References	Resp. failure	ARDS	Coma or Encephalopathy	Shock	Myocardial injury	Heart failure	Coag dysfxn	AKI	Multiorgan dysfxn	Liver dysfxn	IMV	ECMO	Death
Al Kuwari (25)		**X**											
Arslan et al. (23)	**X**		**X**	**X**		**X**	**X**	**X**					
Aykac (26)	**X**	**X**	**X**	**X**	**X**	**X**	**X**	**X**					
Aykac (27)	**X**	**X**	**X**	**X**	**X**		**X**	**X**	**X**				
Bellino (28)		**X**		**X**					**X**				
Ece (29)	**X**	**X**	**X**	**X**	**X**	**X**	**X**		**X**				
Finelli (30)				**X**	**X**	**X**		**X**		**X**			
Geng (31)				**X**					**X**		**X**		
Giacomet (32)	**X**	**X**		**X**					**X**				
Guo (22)	**X**			**X**									
Guo (33)	**X**			**X**					**X**				
Krajcar (34)		**X**		**X**									
Kushner (35)				**X**					**X**		**X**		
Lazzerini (36)									**X**		**X**		
Oh (37)											**X**	**X**	**X**
Otto (38)											**X**		
Ozenen (39)	**X**	**X**	**X**	**X**	**X**	**X**	**X**	**X**					
Parri (40)	**X**			**X**					**X**		**X**		
Parri (41)			**X**	**X**	**X**	**X**	**X**	**X**	**X**		**X**		
Preston (42)											**X**		**X**
Rabha (43)				**X**					**X**		**X**		
Saraiva (44)		**X**		**X**									
Sharma (45)		**X**		**X**					**X**				
Siddiqui (46)	**X**			**X**					**X**				
Soysal (47)	**X**	**X**	**X**	**X**	**X**	**X**	**X**	**X**					
Yanover (48)													**X**
Yilmaz (49)	**X**	**X**	**X**	**X**	**X**		**X**	**X**					

*ARDS, acute respiratory distress syndrome; Coag dysfxn, coagulation dysfunction; AKI, acute kidney injury; Multiorgan dysfxn, multiorgan dysfunction; IMV, invasive mechanical ventilation; ECMO, extracorporeal membrane oxygenation. Specific criteria defining each outcome varied across included studies.*

The 2020 World Bank Lending Status classification of countries was employed and countries were classified as low-middle income (low-income, low-middle income, and upper-middle income) or high-income (14). Studies reporting data from more than one country had the data extracted separately for each country when possible; if that was not possible, the study was excluded from the economic analyses.

We divided the pandemic into an “early” (until May 31st, 2020) and “mid” (June 1, 2020 to May 28, 2021) pandemic periods. These terms and dates reflect our study period and the fact that the pandemic remains ongoing and recent data are not included in our study. However, as children were frequently admitted to hospital and ICU simply due to a lack of knowledge and comfort with SARS-CoV-2 infection in children during the earliest days of the pandemic, we felt such an analysis was important.

### Risk of Bias Assessment

Studies were assessed for RoB by two independent reviewers (MS, KL-K, AK, or NL) using a modified version of the Newcastle-Ottawa Risk of Bias Assessment Tool for cross-sectional and cohort studies (see Supplementary Table 2 for scoring criteria for each domain) (15). Studies could receive a maximum score of 9 points over the following domains: 1. Representativeness of exposed cohort (2 points); 2. Ascertainment of exposure (1 point); 3. Presence of outcome before exposure (2 points); 4. Assessment of outcome (1 point); 5. Type of data collection (1 point); 6. Length of follow-up (1 point); and 7. Completeness of follow-up (1 point). We classified studies with ≤7 points as high RoB and studies with ≥8 points as low RoB (15).

### Statistical Analysis

Proportions for our primary outcomes were calculated based on the number of children experiencing the outcome divided by the total number of children for whom outcome data were available. Forest plots were generated and visually inspected to identify outliers which were then re-reviewed by two independent reviewers to confirm eligibility, data extraction, and identify potential sources of heterogeneity (see Supplementary Material). Risk estimates were pooled across studies using the *metaprop* command in STATA which uses the DerSimonian and Laird random effects model after Freeman-Tukey double arcsine transformation (16–18). Confidence intervals for the effect size of individual studies were generated using the Wilson score test, and those for pooled estimates were generated using the Wald test (16). We used random effects models to reflect the variations observed across studies and assessed between-study heterogeneity using the *I*^2^ statistic with values ≥75% suggesting considerable heterogeneity (19). The Chi^2^ statistic was computed to compare pooled effect size across groups. Statistical tests were 2-sided, and *P* < 0.05 was considered statistically significant. Data analyses were performed using Stata, version 16.1 (StataCorp LLC).

### Sensitivity Analysis

Given the heterogeneity of both the availability and indications for COVID-19 testing across studies, we performed two sensitivity analysis. First, our analysis of population-based studies was repeated excluding studies without clearly identified testing criteria. We compared these results with our primary analysis. For our second sensitivity analysis, we removed all studies that explicitly stated that they included asymptomatic individuals.

## Results

### Literature Search

**FIGURE 1:**
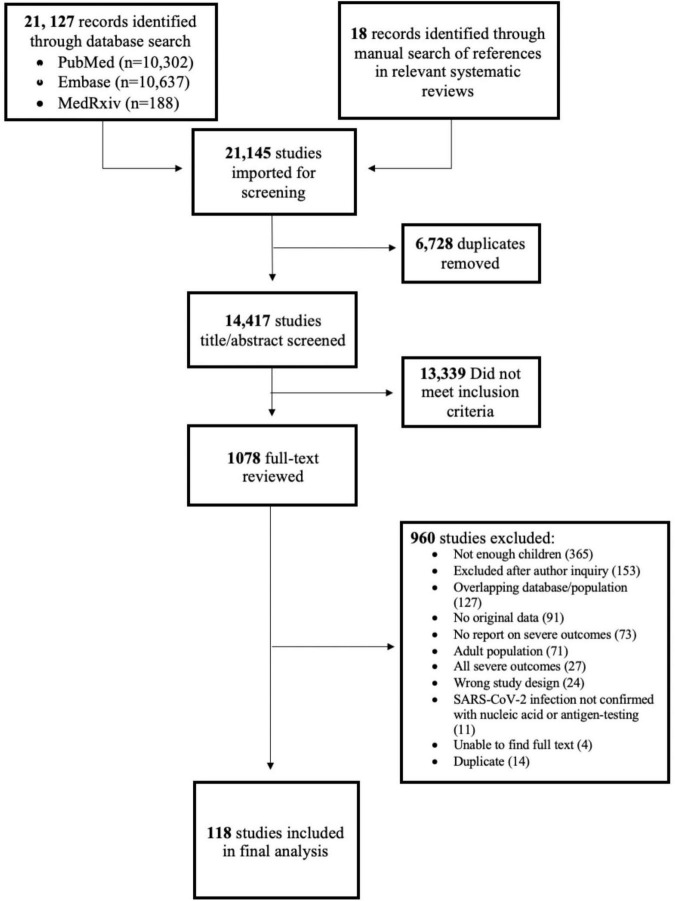
PRISMA diagram.

21,127 citations were identified through database searches and 18 additional records were added manually after reviewing relevant systematic review reference lists. After removal of duplicates, 14,417 studies entered title/abstract screening, 1,078 studies progressed to full-text review, and 118 studies were included in our analysis (Figure 1).

### Study Characteristics

The 118 included studies included data from 68 countries and 3,324,851 children (Supplementary Table 3). Studies most frequently were from the United States (*N* = 23), Italy (*N* = 11), and Turkey (*N* = 9). Forty-eight (41%) studies were population-based and 70 (59%) were hospital-based; of the latter group, 31 (44%) reported on inpatients only, and 39 (56%) reported on outpatients, emergency department (ED), and inpatients. The most reported outcome was death (91/118; 77%), followed by ICU admission (66/118; 56%), hospitalization (52/118; 44%), and severe outcomes (38/118; 32%). Invasive mechanical ventilation (IMV), non-invasive ventilation (NIV), and severe outcomes were reported by 38 (32%), 14 (12%), and 23 (19%) studies, respectively.

### Risk of Bias

**FIGURE 2:**
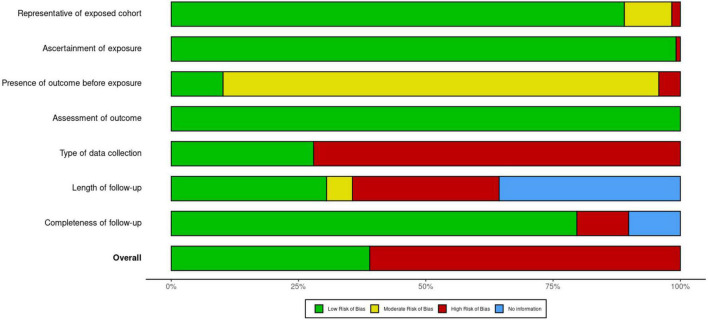
Summary of risk of bias judgments for included studies.

Sixty-one percent (72/118) of studies were classified as high RoB; 46 (39%) were low RoB (Figure 2 and Supplementary Figure 1). Domains with the highest RoB were “Presence of Outcome Before Exposure” (106 studies were biased toward including children who were more symptomatic/severe than the average SARS-CoV-2 infected child in the community), and “Type of Data Collection” (85 studies were cross-sectional or retrospective and lacked a follow-up component).

### Primary Analysis

#### Population-Based Studies

**FIGURE 3:**
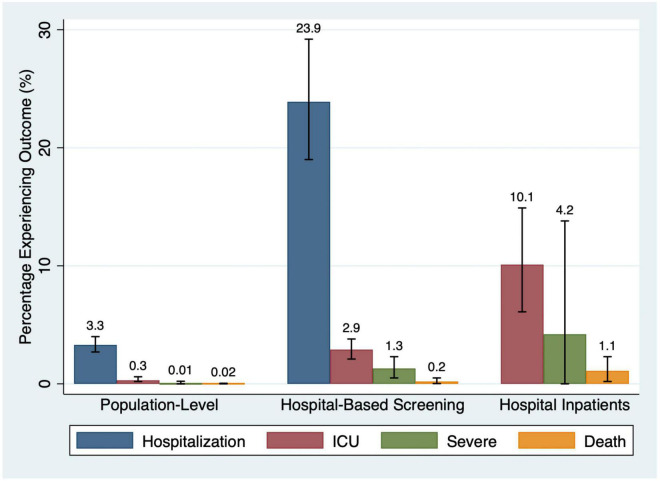
Percentage of participants experiencing outcomes by study type.

**FIGURE 4:**
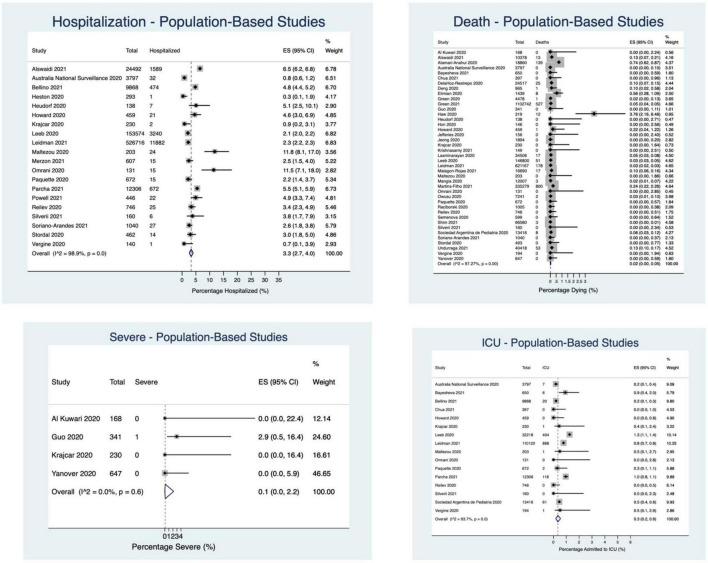
Percentage of children experiencing outcomes in population-based studies.

Of the population-based studies (*N* = 48), 20 reported hospitalization (736,480 participants), 16 reported ICU admission (185,567 participants), 41 reported the proportion of children dying (*N* = 2,472,217 participants), and four reported “severe” outcomes (*N* = 1,386 participants). 3.3% of children were hospitalized (95%CI: 2.7–4.0%; *I*^2^ = 99%), 0.3% of children were admitted to the ICU (95%CI: 0.2–0.6%; *I*^2^ = 94%), 0.1% experienced a severe outcome (95%CI: 0.00–2.2%; *I*^2^ = 0%), and 0.02% of children died (95%CI: 0.001–0.05%; *I*^2^ = 97%) (Figures 3, 4).

#### Hospital-Based Screening Studies

**FIGURE 5:**
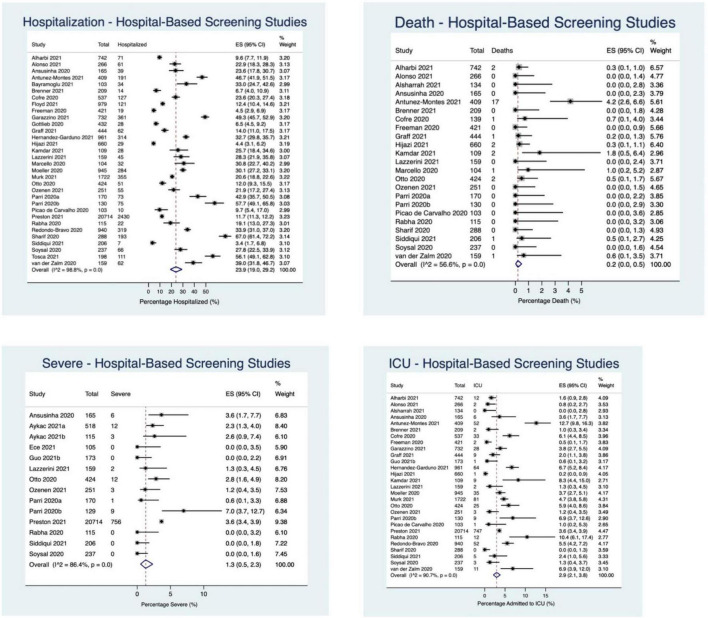
Percentage of children experiencing outcomes in hospital-based screening studies.

In the hospital-based screening studies (*N* = 39), 32 reported hospitalization (*N* = 34,034 participants), 28 reported ICU admission (32,355 participants), 23 reported death (6,044 participants), and 14 reported “severe” outcomes (*N* = 23,481 participants). 23.9% of children required hospitalization (95%CI: 19.0–29.2%; *I*^2^ = 99%), 2.9% (95%CI: 2.1–3.8%; *I*^2^ = 91%) were admitted to the ICU, 1.3% had a severe outcome (95%CI: 0.5–2.3%; *I*^2^ = 86%), and 0.2% died (95%CI: 0.02–0.5%; *I*^2^ = 57%) (Figures 3, 5).

#### Inpatient Only Studies

**FIGURE 6:**
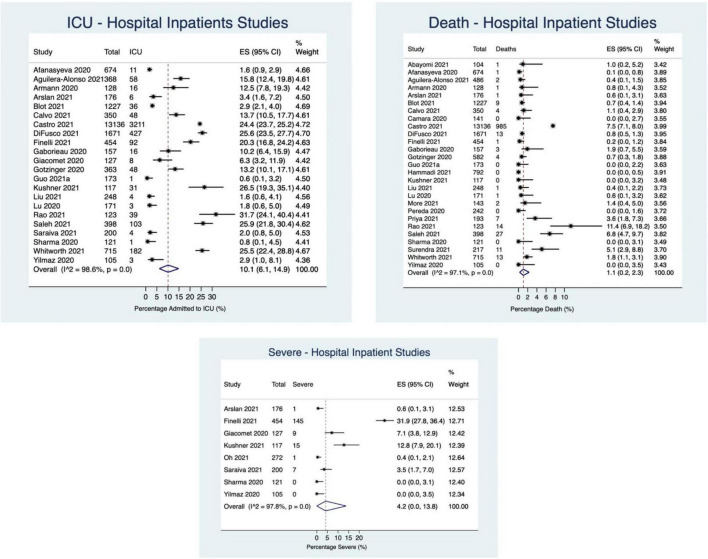
Percentage of children experiencing outcomes in hospital inpatient studies.

Thirty-one studies reported on hospital inpatients only. Of these studies, ICU admission was reported in 22 studies (21,202 participants), death was reported in 27 studies (23,044 participants), and “severe” outcome was reported in 8 studies (*N* = 1,572 participants). 10.1% of patients were admitted to the ICU (95%CI: 6.1–14.9%; *I*^2^ = 99%), 4.2% of patients experienced a “severe” outcome (95%CI: 0.0–13.8%; *I*^2^ = 98%), and 1.1% of patients died (95%CI: 0.2–2.3%; *I*^2^ = 97%) (Figures 3, 6).

#### Level of Respiratory Support

Fourteen and thirty-eight studies reported the proportion of children requiring non-invasive and invasive ventilation, respectively. In the population-based studies, 0.1% of children required invasive mechanical ventilation (95% CI: 0.0–0.3; *I*^2^ = 53%). In hospital-based screening studies, 1.7% of children required non-invasive ventilatory support (95% CI: 0.5–3.5; *I*^2^ = 83%), and 0.8% required invasive mechanical ventilation (95% CI: 0.3–1.4%; *I*^2^ = 84%). Studies reporting on hospital inpatients only described 4.6% of children requiring non-invasive ventilation (95% CI: 2.2–7.7%; *I*^2^ = 80%) and 3.4% requiring invasive mechanical ventilation (95% CI: 1.7–5.7%; *I*^2^ = 93%).

### Comparative Analyses

#### Risk of Bias

**FIGURE 7:**
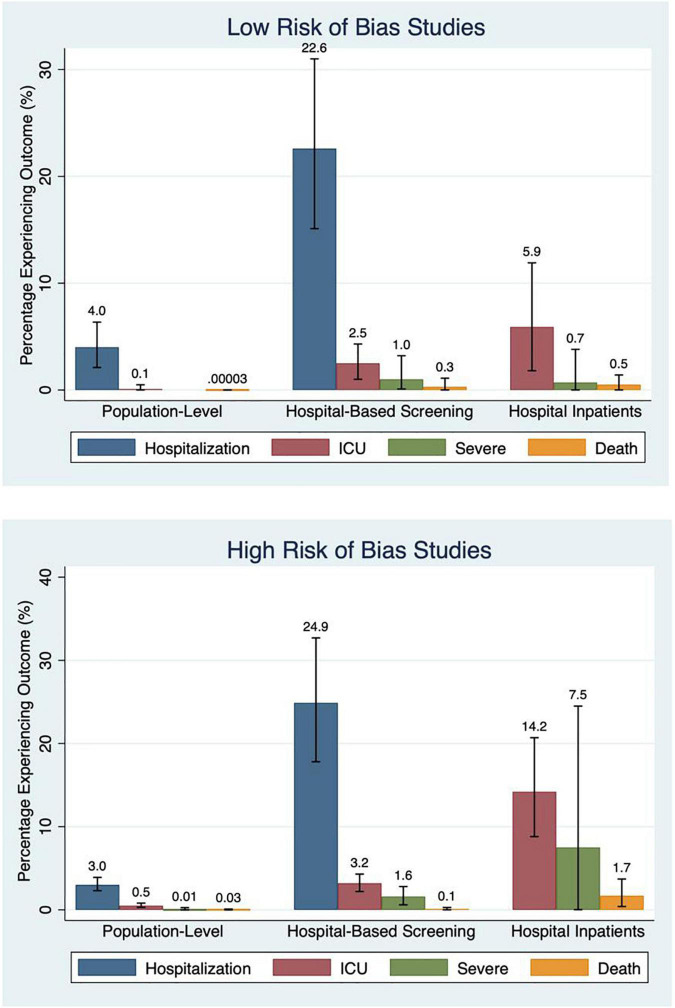
Percentage experiencing outcomes by risk of bias.

Low RoB studies, compared with high RoB studies, had lower point estimate event rates across all study outcomes and populations, except for death in hospital-based studies and hospital admission in population-based studies. This difference was most pronounced for the outcome of ICU admission in population-based (high RoB – 0.5%; low RoB – 0.1%; *P* = 0.03) and hospitalized inpatients studies (high RoB – 14.2%; low RoB – 5.9%; *P* = 0.04) (Table 2 and Figure 7).

**TABLE 2:** Percentage of participants experiencing outcome based on risk of bias and pandemic timing.

	Hospitalization (52 studies; 770,514 participants)	Intensive care unit admission (66 studies; 239,124 participants)	Severe outcome (26 studies; 26,439 participants)	Death (91 studies; 2,501,305 participants)
**Subgroups by risk of bias**				
**Population-based studies** **(48 studies; 3,261,421 participants)**				
Low risk of bias (14 studies; 36,648 participants)	**4.0%** (2.1–6.3%) *I*^2^ = 89%	**0.1%** (0.00–0.5%) *I*^2^ = 56%	**N/A** (Insufficient studies)	**0.00004%** (0.00–0.01%) *I*^2^ = 0%
High risk of bias (34 studies; 3,224,733 participants)	**3.0%** (2.3–3.9%) *I*^2^ = 99%	**0.5%** (0.3–0.8%) *I*^2^ = 96%	**0.1%** (0.00–2.6%) *I*^2^ = 0%	**0.03%** (0.01–0.07%) *I*^2^ = 98%
**Hospital-based screening studies** **(39 studies; 39,685 participants)**				
Low risk of bias (18 studies; 10,927 participants)	**22.6%** (15.1–31.0%) *I*^2^ = 98%	**2.5%** (1.0–4.6%) *I*^2^ = 93%	**1.0%** (0.0–3.2%) *I*^2^ = 81%	**0.3%** (0.00–1.1%) *I*^2^ = 74%
High risk of bias (21 studies; 28,758 participants)	**24.9%** (17.8–32.7%) *I*^2^ = 99%	**3.2%** (2.2–4.3%) *I*^2^ = 89%	**1.6%** (0.6–2.8%) *I*^2^ = 84%	**0.1%** (0.01–0.3%) *I*^2^ = 0%
**Hospital inpatients studies** **(31 studies; 23,745 participants)**				
Low risk of bias (14 studies; 3,965 participants)	N/A	**5.9%** (1.8–11.9%) *I*^2^ = 96%	**0.7%** (0.00–3.77%) *I*^2^ = 78%	**0.5%** (0.003–1.4) *I*^2^ = 86%
High risk of bias (17 studies; 19,780 participants)	N/A	**14.2%** (8.8–20.7%) *I*^2^ = 99%	**7.54%** (0.02–24.5%) *I*^2^ = 98%	**1.7%** (0.4–3.7) *I*^2^ = 98%
**Subgroups by pandemic timing**				
**Population-based studies** **(48 studies; 3,261,421 participants)**				
Early pandemic (before May 31st, 2020) (18 studies; 38,701 participants)	**3.4%** (1.8–5.2%) *I*^2^ = 76%	**0.04%** (0.0–0.26%) *I*^2^ = 3%	**0.1%** (0.00–3.0%) *I*^2^ = 0%	**0.05%** (0.0–0.3%) *I*^2^ = 92%
Mid-pandemic (after May 31st, 2020) (30 studies; 3,222,720 participants)	**3.3%** (2.6–4.1%) *I*^2^ = 99%	**0.5%** (0.3–0.7%) *I*^2^ = 95%	**N/A** (Insufficient studies)	**0.02%** (0.001–0.05%) *I*^2^ = 98%
**Hospital-based screening studies** **(39 studies; 39,685 participants)**				
Early pandemic (before May 31st, 2020) (13 studies; 5,235 participants)	**36.3%** (27.8–45.4) *I*^2^ = 97%	**2.3%** (1.1–4.1%) *I*^2^ = 88%	**1.3%** (0.09–3.7%) *I*^2^ = 79%	**0.006%** (0.0–0.3%) *I*^2^ = 0%
Mid-pandemic (after May 31st, 2020) (26 studies; 34,450 participants)	**18.9%** (13.9–24.5%) *I*^2^ = 99%	**3.2%** (2.1–4.5%) *I*^2^ = 92%	**1.2%** (0.3–2.6%) *I*^2^ = 88%	**0.3%** (0.04–0.7%) *I*^2^ = 67%
**Hospital inpatients studies** **(31 studies; 23,745 participants)**				
Early pandemic (before May 31st, 2020) (12 studies; 3,754 participants)	N/A	**5.6%** (2.8–9.3%) *I*^2^ = 93%	**1.8%** (0.0–6.3%) *I*^2^ = 87%	**0.6%** (0.4–1.0) *I*^2^ = 0%
Mid-pandemic (after May 31st, 2020) (18 studies; 19,887 participants)	N/A	**14.6%** (9.4–20.6%) *I*^2^ = 98%	**6.0%** (0.0–22.4%) *I*^2^ = 98%	**1.3%** (0.2–3.3) *I*^2^ = 98%

*Early pandemic represents recruitment ending prior to May 31, 2020; mid-pandemic included participants recruited after that date.*

#### Economic Status

**FIGURE 8:**
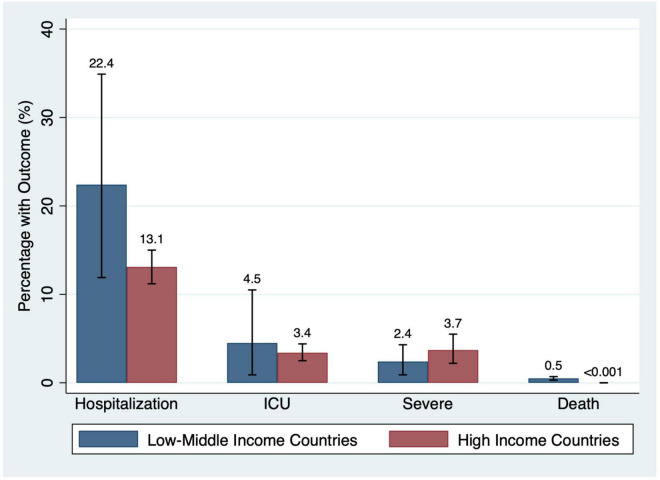
Percentage of participants with outcomes by country income status.

Studies reporting data from low-middle income countries (LMIC; *N* = 59) had higher point estimate event rates of hospitalization (22.4% in LMIC vs. 13.1% in high-income countries; *p* < 0.001), ICU admission (4.5% in LMIC vs. 3.4% in high-income countries; *p* = 0.74), and death (0.5% in LMIC vs. <0.001% in high-income countries; *p* < 0.001) than studies from high-income countries (*n* = 73) (Figure 8).

#### Pandemic Timing

43 (36%) studies had a recruitment end date prior to May 31st, 2020 (i.e., early pandemic) and 75 (64%) were classified as mid-pandemic. Although studies that included children tested for SARS-CoV-2 infection in hospitals had greater proportions hospitalized in the early pandemic period (36.3 vs. 18.9%; *p* = 0.004), among hospitalized children the proportions admitted to ICU and experiencing severe outcomes were higher in the mid-pandemic period (14.6 and 3.2% vs. 5.6 and 2.3%; *P* = 0.005 and *P* = 0.04) (Table 2).

### Sensitivity Analyses

Of the 49 population-based studies, 25 (51%) did not report the indications for testing. Excluding these studies did not meaningfully alter our estimates for hospitalization (3.6% with asymptomatic excluded, *I*^2^ = 88% vs. 3.3% overall, *I*^2^ = 99%), ICU admission (0.1%, *I*^2^ = 52% vs. 0.3%, *I*^2^ = 97% overall), severe outcome (0.09%, *I*^2^ = 0% vs. 0.1%, *I*^2^ = 0% overall), or death (0.02%, *I*^2^ = 76% vs. 0.02%, *I*^2^ = 99% overall). However, this sensitivity analysis did reduce the heterogeneity of the estimates, as evidenced by lower *I*^2^ values.

Removing studies with asymptomatic children (*N* = 29) raised our estimate of both population-based hospitalization and death marginally (3.4 vs. 3.3% and 0.03 vs. 0.02%, respectively). ICU rates remained constant (0.3 vs. 0.3%), and our severe outcome estimate shifted lower (0.00 vs. 0.1%).

## Discussion

In this meta-analysis, we synthesized data from 118 studies and over 3 million SARS-CoV-2-infected children. We found that hospitalization, ICU admission, and severe outcome occur in 3.3, 0.3, and 0.1% of children recruited from population-based settings and 23.9, 2.9, and 1.3% of those recruited in hospital-based venues, respectively. Among SARS-CoV-2 positive hospitalized inpatients, 10.1% of children are admitted to the ICU and 4.2% experience a severe outcome. Death occurred in 0.02% of children in population-based samples, 0.2% in hospital-based screening (i.e., ED) and 1.1% of hospital inpatients. Lower event rates were reported in low RoB studies and those conducted in high-income countries. While hospitalization occurred more frequently early in the pandemic, as it evolved, ICU admissions and severe outcomes occurred in a greater proportion of inpatient children. There was significant between-study heterogeneity (*I*^2^ > 75%) for almost all pooled estimates, including in subgroup analyses.

Meta-analyses, published in early 2021, reported that the risk of severe outcomes for SARS-CoV-2 infected children was between 4 and 7%, with significant inter-study heterogeneity (*I*^2^ > 80%) (9–11). Our meta-analysis, which we believe is the first to systematically analyze sources of heterogeneity within estimates through sub-group analysis, clarifies that the risk of severe outcomes is primarily limited to children who are hospitalized and does not reflect the risk in the broader population of SARS-CoV-2 infected children. A much lower percentage of children tested in a community setting experienced severe outcomes (0.1%) compared to hospital screening (e.g., ED; 1.3%) and inpatients (4.2%). We additionally determined that study RoB, country income status, and pandemic timing were associated with the reported risk of severe outcomes.

Our findings of risk within populations and settings aligns with prior reports. A meta-analysis that examined pediatric inpatients estimated that 11% of children are admitted to an ICU, and 2.4% die (20); these results are similar to our estimates of 10.1 and 1.1%, respectively. Similarly, in our community analysis, 0.3% of children were admitted to an ICU and 0.02% died, closely mirroring CDC estimates of 0.7 and 0.03%, respectively (3). Our hospitalization rate, however, was higher than predicted; while Public Health Agency of Canada data indicates that 0.5% of SARS-CoV-2-infected children require hospitalization (8), our meta-analysis revealed a rate over 6-fold higher (3.3%). There are several possible explanations for this discrepancy. Our data are highly heterogeneous (*I*^2^ = 99%) and the overall point estimate is driven by a few studies with small numbers of children (Supplementary Material). Asymptomatic or mildly symptomatic children may also be underrepresented in our analysis, given that these patients were less likely to seek testing, and/or may have been ineligible for testing, depending on the timing and setting of infection. As shown in our sensitivity analysis, removing studies with unclear testing indications reduced the heterogeneity of the estimate (*I*^2^ = 88%). Additionally, we found that hospitalization rates were higher early in the pandemic, particularly among those tested in hospitals (36.3 vs. 18.9%). This likely reflects the concerns emerging from the early and limited pediatric specific data.

We found that studies reporting outcomes prior to May 31, 2020, had higher hospitalization rates. During this time, there was a great deal of uncertainty regarding the COVID-19 clinical course in pediatric patients and many children with SARS-CoV-2 infection were hospitalized, irrespective of disease severity (21), particularly in countries such as China and Turkey (22, 23). Among inpatients however, we found that a higher proportion experienced ICU admission (14.6 vs. 5.6%) and severe outcome (6.0 vs. 1.8%) during the mid-pandemic period. Although this likely reflects the fact that those hospitalized as the pandemic progressed had more severe symptoms, there is evidence that the Delta variant of concern (VoC) increased the risk of hospitalization in children <10 years by a factor of 2.5 and that younger patients had a greater relative increased risk of death compared with older individuals (24).

Major limitations of our meta-analysis are the heterogeneity of our estimates, and the applicability of our results to the current state of the pandemic. The heterogeneity is a by-product of the variations in study quality, patient population, local testing, and management strategies, all of which evolved over time. Our low RoB subgroup analyses are likely more reflective of the true percentage of children experiencing severe outcomes. Secondly, our meta-analysis only includes data published through May 28, 2021. Since that date, the pandemic has changed with the emergence of the Omicron VoC. Additionally, as vaccination was not yet available for children during our study period, we could not incorporate its effects into our analysis. Finally, as a separate search strategy would have been required to identify studies reporting on the incidence of MIS-C, we were unable to report on this outcome, which is nonetheless important, as it accounts for a large proportion of pediatric ICU admissions and morbidity in this population.

## Conclusion

This meta-analysis, which included 118 studies and over 3 million SARS-CoV-2 infected children, demonstrates that although hospitalization was common early in the pandemic, especially in LMICs, severe outcomes and death were uncommon, occurring in 0.1 and 0.02% of infected children in population-based samples. Estimates of severe outcomes vary by study population, RoB, economic status of the country, and pandemic timing. Our meta-analysis merges disparate and highly heterogeneous risk estimates in the literature to provide clinically meaningful pediatric COVID-19 prognostic data.

## Data Availability Statement

The original contributions presented in the study are included in the article/Supplementary Material, further inquiries can be directed to the corresponding author.

## Author Contributions

MS contributed to study concept and design, title/abstract and full text screening, data extraction, data analysis, manuscript drafting, and manuscript critical revision. AK contributed to title/abstract and full text screening, data extraction, and manuscript critical revision. KL-K contributed to study concept and design, title/abstract and full text screening, and data extraction. NL contributed to data extraction and manuscript critical revision. DL contributed to study concept and design, search strategy, and manuscript critical revision. AF conceptualized and designed the study, assisted with title/abstract and full text screening, data extraction and analysis, participated in revising of the manuscript, and supervised the conduct of the study. SF conceptualized and designed the study, assisted with abstract and full-text screening, data extraction and analysis, participated in revising of the manuscript, and supervised the conduct of the study. All authors approved the final manuscript as submitted and agreed to be accountable for all aspects of the work.

## Conflict of Interest

The authors declare that the research was conducted in the absence of any commercial or financial relationships that could be construed as a potential conflict of interest. The handling editor SB declared past collaboration with one of the authors SF.

## Publisher’s Note

All claims expressed in this article are solely those of the authors and do not necessarily represent those of their affiliated organizations, or those of the publisher, the editors and the reviewers. Any product that may be evaluated in this article, or claim that may be made by its manufacturer, is not guaranteed or endorsed by the publisher.

## Supplementary Material

Supplementary Figure 1 Risk of bias assessment for included studies.

Supplementary Table 1 Search strategy.

Supplementary Table 2 Adapted newcastle-ottawa risk of bias scale for cross-sectional and cohort studies.

Supplementary Table 3 Selected characteristics of included studies.

Supplementary Text Description of major outliers.
