# Supplementary material for: Severe Outcomes Associated With SARS-CoV-2 Infection in Children: A Systematic Review and Meta-Analysis
Source: Front Pediatr. 2022 Jun 9;10:916655. doi: 10.3389/fped.2022.916655 (PMC9218576; doi:10.3389/fped.2022.916655)

eFigure 1: Risk of Bias Assessment of Included Studies

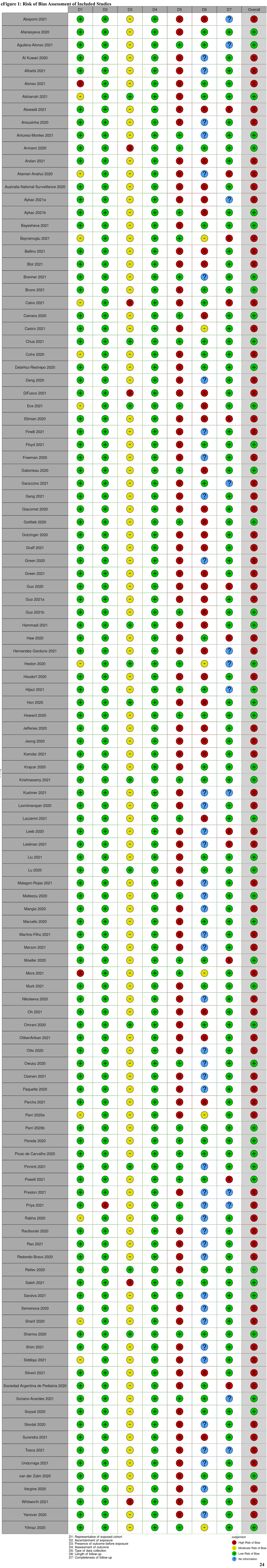

Supplement: Supplementary Figure 1 — Risk of bias assessment for included studies. [file Image_1.PDF]
