# Supplementary material for: Severe Outcomes Associated With SARS-CoV-2 Infection in Children: A Systematic Review and Meta-Analysis
Source: Front Pediatr. 2022 Jun 9;10:916655. doi: 10.3389/fped.2022.916655 (PMC9218576; doi:10.3389/fped.2022.916655)
Supplement: Supplementary Table 1 — Search strategy. [file Table_1.DOCX]

**eTable 1a:** Search Strategy (PUBMED)

**Database:** PUBMED

**Search Date:** May 28, 2021

| **Item** | **Subject Headings and Specific Terms** | **# Records** |
| --- | --- | --- |
| 1 | COVID-19[MeSH] OR SARS-CoV-2[MeSH] | 81,740 |
| 2 | “coronavirus disease 2019”[tiab] OR covid-19[tiab] OR covid19[tiab] OR 2019-ncov[tiab] OR n-cov[tiab] OR “2019 novel coronavirus”[tiab] OR “severe acute respiratory syndrome coronavirus 2”[tiab] OR sars-cov-2[tiab] OR sarscov2[tiab] OR sars-cov2[tiab] | 131,761 |
| 3 | 1 OR 2 | 136,105 |
| 4 | Infant[MeSH] or Child[MeSH] or Adolescent[MeSH] | 3,673,325 |
| 5 | newborn*[tiab] or baby[tiab] or babies[tiab] or neonat*[tiab] or infant*[tiab] or child*[tiab] or teen*[tiab] or adolescen*[tiab] or pediatr*[tiab] or paediatr*[tiab] | 2,391,235 |
| 6 | 4 OR 5 | 4,375,224 |
| 7 | 3 AND 6 | 13,937 |
| 8  *(exclusion)* | Case Reports[pt] OR Comment[pt] OR Editorial[pt] OR Guideline[pt] OR Practice Guideline[pt] OR News[pt] OR Newspaper Article[pt] OR Review[pt] OR Systematic Review[pt] OR Meta-Analysis[pt] | 6,452,021 |
| 9 | 7 NOT 8 | 10,352 |
| 10 *(exclusion)* | Animals[MeSH] NOT Humans[MeSH] | 4,835,343 |
| 11 | 9 NOT 10 | **10,347** |
| 12  *(date limit)* | December 1^st^ 2019 to May 28^th^ 2021 | **10,288** |

**eTable 1b:** Search Strategy (EMBASE via Ovid)

**Database:** Embase (1974-Current) via Ovid

**Search Date:** May 28, 2021 (Ovid searches until May 27, 2021)

| **Item** | **Subject Headings and Specific Terms** | **# Records** |
| --- | --- | --- |
| 1 | exp coronavirus disease 2019/ or exp Severe acute respiratory syndrome coronavirus 2/ | 119,590 |
| 2 | (coronavirus disease 2019 or covid-19 or covid19 or 2019-ncov or n-cov or 2019 novel coronavirus or severe acute respiratory syndrome coronavirus 2 or sars-cov-2 or sarscov2 or sars-cov2).ti,ab,kw. | 130,929 |
| 3 | 1 OR 2 | 141,853 |
| 4 | exp child/ or exp adolescent/ | 3,518,392 |
| 5 | (newborn* or baby or babies or neonat* or infant* or child* or teen* or adolescen* or pediatr* or paediatr*).ti,ab,kw. | 2,917,742 |
| 6 | 4 OR 5 | 4,344,526 |
| 7 | 3 AND 6 | 13,697 |
| 8  *(exclusion)* | Limit 7 to (books or chapter or conference abstract or conference paper or "conference review" or editorial or "review") | 3048 |
| 9 | 7 NOT 8 | 10,649 |
| 10  *(exclusion)* | Limit 9 to (animals and animal studies) | 11 |
| 11 | 9 NOT 10 | 10,638 |
| 12  *(date limit)* | Limit 11 to yr="2019 -Current" | **10,637** |
