# Supplementary material for: Severe Outcomes Associated With SARS-CoV-2 Infection in Children: A Systematic Review and Meta-Analysis
Source: Front Pediatr. 2022 Jun 9;10:916655. doi: 10.3389/fped.2022.916655 (PMC9218576; doi:10.3389/fped.2022.916655)
Supplement: Supplementary Table 2 — Adapted newcastle-ottawa risk of bias scale for cross-sectional and cohort studies. [file Table_2.DOCX]

**eTable 2:** Adapted Newcastle-Ottawa Risk of Bias Scale for Cross-Sectional and Cohort Studies

| **SELECTION** | | | **OUTCOME** | | | |
| --- | --- | --- | --- | --- | --- | --- |
| **Representativeness of sample**  **(2 points)** | **Ascertainment of exposure**  **(1 point)** | **Demonstration that outcome of interest was not present at baseline**  **(2 points)** | **Assessment of outcomes**  **(1 point)** | **Type of data collection**  **(1 point)** | **Was follow-up long enough for outcomes occur?**  **(1 point)** | **Adequacy of follow up of cohorts**  **(1 point)** |
| **0 points –** Highly selected group of users.  **1 point:** Somewhat representative of the average exposed SARS-CoV-2 infected child  **2 points:** Truly representative of the average exposed SARS-CoV-2 infected child | **0 points –** Written self-report by participant or caregiver; minority NAT confirmed, or majority antigen  **1 point:** Majority are NAT confirmed via secure record or case-report form linked to secure records.  . | **1 point –** Some severe outcomes were likely present initially  **2 points –** No severe outcomes present at baseline.  **0 points –** If >50% of children were hospitalized, or >10% were admitted to intensive care. | **0 points –** No description of outcome assessment.  **1 point –** Medical record linkage or mixed medical record linkage with participant/ caregiver interview, independent blind assessment, or self-report of participants/caregivers | **0 points –** Passive: data is extracted only from medical records or registries. There is no contact with the participants.  **1 point –** Active (standardized follow-up with some aspect of collecting information from the participants themselves) | **0 points –** Data was collected for a period of <4 days, or until discharge.  **1 point –** Yes, active or medical record data collection was reported between 4-30 days after confirmation of SARS-CoV-2 infection. | **0 points –** No statement on missing data, >20% missing data, or indication that only participants with complete outcome data were included.  **1 point –** <20% of data missing in follow-up |

**We rated studies with ≤7 points as high risk of bias, and those with ≥8 points as low risk of bias
