# Supplementary material for: Severe Outcomes Associated With SARS-CoV-2 Infection in Children: A Systematic Review and Meta-Analysis
Source: Front Pediatr. 2022 Jun 9;10:916655. doi: 10.3389/fped.2022.916655 (PMC9218576; doi:10.3389/fped.2022.916655)
Supplement: Supplementary Table 3 — Selected characteristics of included studies. [file Table_3.DOCX]

**eTable 3:** Selected Characteristics of the Included Studies.

| Author, Year | Country | Study Population | Age Range | Number of Children | Outcomes | Severe Outcome Definition |
| --- | --- | --- | --- | --- | --- | --- |
| Abayomi, 2021^1^ | Nigeria | Inpatients | <20 | 121 | Death |  |
| Afanasyeva, 2020^2^ | Russia | Inpatients | <18 | 674 | ICU; Severe Outcome; IMV; Death | None provided |
| Aguilera-Alonso, 2021^3^ | Spain | Hospital outpatients/inpatients | <18 | 537 | Hospitalization; ICU; High-flow O_2_; IMV; Inotropes; Death |  |
| Al Kuwari, 2020^4^ | Qatar | Database - mixed settings | <18 | 168 | Severe Outcome; Critical Outcome; Death | **Severe –** cough or difficulty in breathing plus one of: central cyanosis or SpO_2_<90%, severe respiratory distress, or signs of pneumonia with a general danger sign: inability to breastfeed or drink, lethargy, unconscious or convulsions  **Critical –** ARDS |
| Alharbi, 2020^5^ | Saudi Arabia | Hospital outpatients/inpatients | <15 | 742 | Hospitalization; ICU; Shock; Inotropes; Heart Failure; Mechanical Ventilation; Death |  |
| Alonso, 2021^6^ | USA | Database of patients with T1DM - mixed settings | <19 | 226 | Hospitalization; ICU; ECMO; Respiratory failure; Mechanical ventilation; Death |  |
| Alsharrah, 2021^7^ | Kuwait | Inpatients | <19 | 134 | ICU; Severe Outcome; Death | Adolescent or adult with clinical signs of pneumonia (fever, cough, dyspnea) plus one of the following: RR > 30 breaths/min;  severe respiratory distress; or SpO2 < 90% on room air. Child with clinical signs of pneumonia (cough or difficulty in breathing)  + at least one of the following: Central cyanosis or SpO2 < 90%; severe respiratory distress (e.g. fast breathing, grunting, very severe chest indrawing); general danger sign: inability to breastfeed or drink, lethargy or unconsciousness, or  convulsions. |
| Alswaidi, 2021^8^ | Saudi Arabia | Database – mixed settings | <16 | 73,966 | Hospitalization; Death |  |
| Ansusinha, 2020^9^ | USA | Hospital outpatients/inpatients | <20 | 165 | Hospitalization; ICU; Critical Outcome; ETT; BiPAP; Death | Respiratory support or MIS-C |
| Antunez-Montes, 2021^10^ | Mexico, Colombia, Peru, Costa Rica | Database – mixed settings | <19 | 409 | Hospitalization; ICU; Mechanical Ventilation; ECMO; Inotropes; Death |  |
| Armann, 2020^11^ | Germany | Inpatients | <20 | 128 | Hospitalization; ICU; Non-invasive or invasive ventilation; tracheostomy; inotropes; RRT; ECMO; Sepsis; Encephalitis; ARDS; Death |  |
| Arslan, 2021^12^ | Turkey | Inpatients | <19 | 176 | Hospitalization; ICU; Severe Outcome; Critical Outcome; Non-invasive ventilation; Death | **Severe -** Respiratory and gastrointestinal system symptoms  **Critical -** Acute respiratory failure, encephalopathy, shock, heart failure, acute renal failure and coagulopathy |
| Atamari-Anahui, 2020^13^ | Argentina, Bolivia, Brasil, Chile, Colombia, Costa Rica, Ecuador, El Salvador, Guatemala, Honduras, Mexico, Nicaragua, Panama, Paraguay, Peru, Uruguay, Venezuela, Cuba, Haiti, Dominican Republic | Database - mixed settings | <20 | 20,757 | Death |  |
| Aykac, 2021a^14^ | Turkey | Hospital outpatients/inpatients | <18 | 518 | Severe Outcome; Critical Outcome | **Severe -** Dyspnea, central cyanosis, SpO_2_ <92%  **Critical –** ARDS, respiratory failure, shock, encephalopathy, myocardial injury, heart failure, coagulation dysfunction, AKI |
| Aykac, 2021b^15^ | Turkey | Hospital outpatients/inpatients | <18 | 115 | Severe Outcome; Critical Outcome | **Severe -** Progressive respiratory disease, dyspnea, and central cyanosis. **Critical –** ARDS, respiratory failure, shock, and organ dysfunction, including encephalopathy, myocardial injury, coagulation abnormalities, and AKI |
| Bayesheva, 2021^16^ | Kazakhstan | Database – mixed settings | <19 | 650 | ICU; Severe Outcome; Mechanical Ventilation; Death | Respiratory and GI symptoms and dyspnea and SpO_2_ <92% |
| Bayramoglu, 2021^17^ | Turkey | Hospital outpatients/inpatients | <18 | 103 | Hospitalization; Severe Outcome | Hospitalized patients with pneumonia confirmed by physical examination and imaging |
| Bellino, 2021^18^ | Italy | Database – mixed settings | <18 | 9847 | Hospitalization; ICU; Severe Outcome; Critical Outcome | **Severe -** pneumonia, hypoxia, dyspnea, tachypnea requiring hospitalization. **Critical -** severe pneumonia, ARDS, septic shock, multiple organ dysfunction requiring ICU admission |
| Blot, 2021^19^ | France | Inpatients | <18 | 1,277 | ICU; Acute respiratory failure; Septic shock; MI; Death |  |
| Brenner, 2021^20^ | 23 countries (International Registry) | Database of patients with IBD – mixed settings | <19 | 209 | Hospitalization; ICU; Mechanical Ventilation; Death |  |
| Bruno, 2021^21^ | USA | Database – mixed settings | <18 | 4,153 | Severe Outcome | Hospitalization <7 days prior to the testing date with diagnosis of pneumonia, sepsis, respiratory failure, or COVID-19 |
| Calvo, 2021^22^ | Spain | Hospital outpatients/inpatients | <18 | 350 | Hospitalization; ICU; Death |  |
| Camara, 2020^23^ | Guinea | Hospital outpatients/inpatients | <17 | 141 | Death |  |
| Castro, 2021^24^ | Brazil | Database – mixed settings | <20 | 13,136 | ICU; Death |  |
| Chua, 2021^25^ | Hong Kong | Community Testing Centre | <19 | 397 | ICU; Death |  |
| Cofre, 2020^26^ | Chile | Hospital outpatients/inpatients | <15 | 537 | Hospitalization; ICU; Death |  |
| COVID-19 National Incident Room Surveillance Team, 2020^27^ | Australia | Database - mixed settings | <19 | 3,797 | Hospitalization; ICU; Death |  |
| De la Hoz-Restrepo, 2020^28^ | Colombia | Database - mixed settings | <20 | 24,517 | Death |  |
| Deng, 2020^29^ | China | Database – mixed settings | <20 | 965 | Death |  |
| DiFusco, 2021^30^ | USA | Inpatients | <18 | 1,671 | ICU; IMV; Death |  |
| Ece, 2021^31^ | Turkey | Hospital outpatients/inpatients | <19 | 105 | Severe Outcome; Critical Outcome | **Severe -** Dyspnea with central cyanosis, or oxygen saturation <92% with other hypoxia manifestations. **Critical -** ARDS, respiratory failure, shock, encephalopathy, myocardial injury, heart failure, coagulation dysfunction, and organ dysfunction |
| Elimian, 2020^32^ | Nigeria | Database - mixed settings | <21 | 1439 | Death |  |
| Finelli, 2021^33^ | USA | Inpatients | <18 | 454 | ICU; Severe Outcome; Death | Renal insufficiency; kidney failure; suspected sepsis or heart failure; myocardial inflammation; liver dysfunction; cytokine stimulation; CRP >7; ESR >30; D-dimer >1000; Triglycerides >265 |
| Floyd, 2021^34^ | USA | Hospital outpatients/inpatients with asthma | <22 | 979 | Hospitalization |  |
| Freeman, 2020^35^ | USA | Database – mixed settings | <18 | 424 | Hospitalization; ICU; BiPAP; Inotropes; Mechanical Ventilation; Death |  |
| Gaborieau, 2020^36^ | France | Inpatients | <18 | 157 | ICU; Non-invasive ventilation; Mechanical ventilation; ECMO; Myocarditis; Septic Shock; Death |  |
| Garazzino, 2021^37^ | Italy | Database – mixed settings | <18 | 732 | Hospitalization; ICU |  |
| Geng, 2021^38^ | China | Database – mixed settings | <20 | 826 | Severe Outcome | **Severe -** shortness of breath, RR >30, SpO_2_ <93%, and PaO2/FiO2 <300mmHg or radiographic findings of pneumonia progressing more than 50% in 24h **Critical** - dyspnea with mechanical ventilation, shock, multiple organ failure, ICU |
| Giacomet, 2020^39^ | Italy | Inpatients | <18 | 127 | ICU; Severe outcome; Critical outcome; ARDS; Myocardial involvement; Bacteremia; Disordered coagulation; AKI; Liver dysfunction; Myositis; Invasive mechanical ventilation | **Severe** - pneumonia, fever, and cough but without hypoxemia or respiratory distress and oxygen saturation <92% and respiratory distress  **Critical** *–* acute respiratory failure, ARDS, shock, or other life-threatening organ dysfunction |
| Gottlieb, 2020^40^ | USA | Hospital outpatients/inpatients | <19 | 432 | Hospitalization |  |
| Gotzinger, 2020^41^ | Austria, Belgium, Bulgaria, Croatia, Denmark, Estonia, Germany, Greece, Hungary, Ireland, Italy, Lithuania, Norway, Portugal, Slovakia, Slovenia, Spain, Sweden, Switzerland, UK | Hospital outpatients/inpatients | <19 | 582 | Hospitalization; ICU; CPAP; Mechanical Ventilation; ECMO; Inotropes; ARDS; Death |  |
| Graff, 2021^42^ | USA | Hospital outpatients/inpatients | <21 | 444 | Hospitalization; ICU; Respiratory support; Death |  |
| Green, 2020^43^ | Sweden | Database – mixed settings | <20 | 4478 | Death |  |
| Green, 2021^44^ | Italy; England; Israel; Spain; Mexico | Database - mixed settings | <20 | 1,102,742 | Death |  |
| Guo, 2020^45^ | China | Database - mixed settings | <15 | 341 | Severe Outcome; Critical Outcome; Heart Failure; Myocardial Injury; Liver Injury; Shock; AKI; Death | **Severe –** respiratory distress with acute hypoxia. **Critical –** respiratory failure and shock |
| Guo, 2021a^46^ | China | Inpatients | <16 | 173 | ICU; Death |  |
| Guo, 2021b^47^ | China | Hospital outpatients/inpatients | <17 | 173 | ICU; Severe Outcome; Critical Outcome | **Severe -** Any of tachypnea independent of fever or crying; oxygen saturation ≤92%; labored breathing, cyanosis and apnea; lethargy and convulsion; difficulty feeding and signs of dehydration  **Critical -** Respiratory failure requiring mechanical ventilation, shock; other organ failure requiring ICU care |
| Hammadi, 2021^48^ | Iraq | Inpatients | <21 | 792 | Death |  |
| Haw, 2020^49^ | Philippines | Database – mixed settings | <21 | 319 | Death |  |
| Hernandez-Garduno, 2021^50^ | Mexico | Database – mixed settings | <18 | 961 | Hospitalization; ICU; Intubation |  |
| Heston, 2020^51^ | USA | Hospital outpatients/inpatients | <21 | 293 | Hospitalization |  |
| Heudorf, 2020^52^ | Germany | Database - mixed settings | <15 | 138 | Hospitalization; ARDS; Mechanical ventilation; Death |  |
| Hijazi, 2021^53^ | Kingdom of Saudi Arabia | Hospital outpatients/inpatients | <16 | 660 | Hospitalization; ICU; Intubation; Death |  |
| Hon, 2020^54^ | Hong Kong | Database - mixed settings | <19 | 146 | Death |  |
| Howard, 2020^55^ | USA | Hospital outpatients/inpatients | <19 | 459 | Hospitalization; ICU; Mechanical ventilation; ECMO; Death |  |
| Jefferies, 2020^56^ | New Zealand | Database – mixed settings | <20 | 156 | Severe Outcome; Death | Hospitalization and/or death |
| Jeong, 2020^57^ | Korea | Database – mixed settings | <20 | 1,894 | Death |  |
| Kamdar, 2021^58^ | USA | Hospital outpatients/inpatients with hematologic or oncologic diagnoses | <18 | 109 | Hospitalization; ICU; Mechanical Ventilation; ECMO; Death |  |
| Krajcar, 2020^59^ | Croatia | Database – mixed settings | <20 | 230 | Hospitalization; ICU; Severe Outcome; Critical Outcome; ARDS; Sepsis/septic shock; Death | **Severe -** Severe pneumonia. **Critical –** ARDS, sepsis, septic shock |
| Krishnasamy, 2021^60^ | India | Non-hospital care center | <19 | 149 | Death |  |
| Kushner, 2021^61^ | USA | Inpatients | <18 | 117 | ICU; Severe outcome; Critical outcome; Death | **Severe -** New or increased oxygen support from baseline but not positive-pressure ventilation. **Critical -** Patients who received invasive or non-invasive ventilation with sepsis or multiorgan failure |
| Laxminarayan, 2020^62^ | India | Database – mixed settings | <18 | 34,506 | Death |  |
| Lazzerini, 2021^63^ | Italy | Database – mixed settings | <19 | 159 | Hospitalization; ICU; Severe Outcome; Critical Outcome; Non-invasive ventilation; Mechanical Ventilation; Death | **Severe -** Oxygen saturation <92% or difficult breathing or other signs of severe respiratory distress or any other danger sign or need for respiratory support  **Critical –** ICU admission or mechanical ventilation or multi-organ failure  or shock, encephalopathy, myocardial injury or heart failure, coagulation dysfunction, AKI, coma. |
| Leeb, 2020^64^ | USA | Database – mixed settings | <18 | 277,285 | Hospitalization; ICU; Death |  |
| Leidman, 2021^65^ | USA | Database – mixed settings | <18 | 1,222, 023 | Hospitalization; ICU; Death |  |
| Liu, 2021^66^ | China | Inpatients | <18 | 248 | ICU; Severe Outcomes; IMV; Cardiac injury; Liver dysfunction; Renal injury; Death | Any of the following criteria: increased respiratory rate; oxygen saturation <92%; hypoxia, assisted breathing cyanosis, or intermittent apnoea; disturbance of consciousness: somnolence, coma, or convulsion; food refusal or feeding difficulty, with signs of dehydration. |
| Lu, 2020^67^ | China | Hospital outpatients/inpatients | <16 | 171 | ICU; Mechanical Ventilation; Lymphopenia; Death |  |
| Malagon-Rojas, 2021^68^ | Colombia | Database – mixed settings | <18 | 16,591 | Death |  |
| Maltezou, 2020^69^ | Greece | Database - mixed settings | <19 | 203 | Hospitalization; ICU: Severe outcome; Death | Admission to ICU or death |
| Mangia, 2020^70^ | Germany | Database – mixed settings | <20 | 12,007 | Death |  |
| Marcello, 2020^71^ | USA | Hospital outpatients/inpatients | <18 | 104 | Hospitalization; Death |  |
| Martins-Filho, 2021^72^ | Brazil | Database – mixed settings | <20 | 335, 279 | Death |  |
| Merzon, 2021^74^ | Israel | Database – mixed settings | <20 | 607 | Hospitalization |  |
| Moeller, 2020^75^ | 35 countries (international registry) | Online | <18 | 945 | Hospitalization; ICU |  |
| More, 2021^76^ | India | Neonate inpatients | <28 days | 143 | Resuscitation at birth; RDS; Septic shock; DIC: Encephalopathy; Ventilation; Inotropes; Death |  |
| Murk, 2021^77^ | USA | Database – mixed settings | <20 | 1722 | Hospitalization; ICU |  |
| Nikolaeva, 2020^78^ | Russia | Database – mixed settings | <18 | 19136 | Hospitalization; Severe Outcome | None provided |
| Oh, 2021^79^ | Korea | Database – mixed settings | <20 | 272 | Severe Outcome; Critical Outcome | **Severe -** Patients receiving oxygen therapy  **Critical –** Ventilation, ECMO, or death |
| Omrani, 2020^80^ | Qatar | Database – mixed settings | <15 | 131 | Hospitalization; ICU; Death |  |
| Otiken Arikan, 2021^81^ | Turkey | Hospital outpatients/inpatients | <18 | 353 | Severe Outcome |  |
| Otto, 2020^82^ | USA | Hospital outpatients/inpatients | <22 | 424 | Hospitalization; ICU; Critical Outcome; Non-invasive ventilation; Mechanical ventilation; ECMO; Vasopressors; Death | Mechanical ventilation |
| Owusu, 2020^83^ | Ghana | Hospital outpatients/inpatients | <18 | 7,241 | Death |  |
| Ozenen, 2021^84^ | Turkey | Hospital outpatients/inpatients | <18 | 251 | Hospitalization; ICU; Severe Outcome; Critical Outcome; O_2_ support; Mechanical ventilation; Death | **Severe -** Respiratory symptoms accompanied by GI symptoms and dyspnea and SpO_2_ <92%  **Critical -** ARDS or respiratory failure, shock, encephalopathy, myocardial injury or heart failure, coagulation dysfunction, AKI |
| Paquette, 2020^85^ | Canada | Database – mixed settings | <19 | 938 | Hospitalization; ICU; Death |  |
| Parcha, 2021^86^ | USA | Hospital outpatients/inpatients | <18 | 12306 | Hospitalization; ICU; IMV |  |
| Parri, 2020a^87^ | Italy | Emergency Room | <19 | 170 | Hospitalization; Severe Outcome; Critical Outcome; Non-invasive ventilation; Mechanical ventilation; Death | **Severe -** Rapid disease progression with tachypnea, hypoxemia, neurologic deterioration, dehydration, myocardial injury, coagulation dysfunction, or rhabdomyolysis. **Critical -** Quick progression of disease with respiratory failure with need for mechanical ventilation, septic shock, or multiple organ failure |
| Parri, 2020b^88^ | Italy | Hospital inpatients/outpatients | <19 | 130 | Hospitalization; ICU; Severe Outcome; Critical Outcome; Non-invasive ventilation; Intubation; Death | **Severe -** Oxygen saturation <92% or difficulty breathing or other signs of respiratory distress or need for respiratory support  **Critical -** Patient in ICU or intubated or multi-organ failure or shock, encephalopathy, myocardial injury or heart failure, coagulation dysfunction, AKI |
| Pereda, 2020^89^ | Cuba | Inpatients | <18 | 242 | Death |  |
| Picao de Carvalho, 2020^90^ | Portugal | Hospital outpatients/inpatients | <18 | 103 | Hospitalization; ICU; Mechanical Ventilation; MIS-C; Death |  |
| Pinninti, 2021^91^ | USA | Inpatients | <21 | 102 | Severe Outcome; Non-invasive ventilation; Invasive ventilation | Pneumonia with hypoxemia requiring ventilatory support, abnormal chest imaging, respiratory failure, shock, or multi-organ dysfunction |
| Powell, 2021^92^ | England | Community Testing Centre | <18 | 446 | Hospitalization |  |
| Preston, 2021^93^ | USA | Hospital outpatients/inpatients | <19 | 20714 | Hospitalization; ICU; IMV; Severe Outcome | Requiring care in an intensive care or step-down unit, requiring invasive mechanical ventilation, or resulting in death. |
| Priya, 2021^94^ | India | Inpatients | <18 | 193 | Death |  |
| Rabha, 2020^95^ | Brazil | Emergency care and inpatients | <18 | 115 | Hospitalization; ICU; Severe Outcome; Critical Outcome; Death | **Severe -** Tachypnea, low oxygen saturation, dyspnea, altered LOC, dehydration, or tomographic imaging with bilateral or multifocal pulmonary infiltrates. **Critical -** ICU with mechanical ventilation, shock, or multiple organ failure. |
| Raciborski, 2020^96^ | Poland | Database – mixed settings | <15 | 1005 | Death |  |
| Rao, 2021^97^ | India | Inpatients | <18 | 123 | ICU; Severe Outcome; MIS-C; Non-invasive ventilation; Invasive ventilation; Vasoactive drugs; Death | Cough or difficulty in breathing, plus one of the following: central  cyanosis or SpO2 <90%; severe respiratory distress; signs of pneumonia with any of the following: inability to  breast-feed or drink, lethargy, unconscious, or convulsions. |
| Redondo-Bravo, 2020^98^ | Spain | Database – mixed settings | <15 | 1038 | Hospitalization; ICU |  |
| Reilev, 2020^99^ | Denmark | Database – mixed settings | <20 | 746 | Hospitalization; ICU; Mechanical Ventilation; Death |  |
| Saleh, 2021^100^ | Egypt | Inpatients | <19 | 398 | ICU; Severe Outcome; Mechanical Ventilation; Acute pancreatitis; DVT: Kawasaki-like disease; Death | ICU admission requiring invasive or non-invasive mechanical ventilation; impending respiratory failure; SpO2 <92% on inspired oxygen >50%; shock; altered mental status. |
| Saraiva, 2021^101^ | Portugal | Inpatients | <19 | 200 | Hospitalization; ICU; Severe Outcome; Critical Outcome; ARDS; Myocarditis; Sepsis; Invasive mechanical ventilation; Inotropes | **Severe -** Pneumonia with severe signs of respiratory distress, severe anorexia, lethargy/decreased level of consciousness, convulsions. **Critical -** ARDS, sepsis, septic shock |
| Semenova, 2020^102^ | Kazakhstan | Database – mixed settings | <18 | 599 | Death |  |
| Sharif, 2020^103^ | United Arab Emirates | Hospital outpatients/inpatients | <17 | 288 | Hospitalization; ICU; Non-invasive ventilation; Invasive ventilation; Sepsis/septic shock; ARDS; Death |  |
| Sharma, 2020^104^ | Nepal | Inpatients | <18 | 121 | ICU; Severe Outcome; Critical Outcome; Ventilation; Inotropes; Death | **Severe -** Pneumonia with at least one danger sign or hypoxia. **Critical -** ARDS, sepsis, or septic shock with evidence of organ dysfunction |
| Shim, 2021^105^ | Canada | Database – mixed settings | <20 | 66,580 | Death |  |
| Siddiqui, 2021^106^ | Turkey | Emergency Room | <18 | 206 | Hospitalization; ICU; Severe Outcome; Critical Outcome; Mechanical Ventilation; Death | **Severe -** Severe respiratory distress, hypoxemia and radiological findings  **Critical –** Respiratory failure, multi-organ failure or shock |
| Silverii, 2021^107^ | Italy | Database – mixed settings | <20 | 160 | Hospitalization; ICU; Death |  |
| Soriano-Arandes, 2021^108^ | Spain | Database – mixed settings | <16 | 1040 | Hospitalization; Death |  |
| Soysal, 2020^109^ | Turkey | Hospital outpatients/inpatients | <18 | 237 | Hospitalization; ICU; Severe Outcome; Critical Outcome; Death | **Severe -** Dyspnea, central cyanosis, SpO_2_<92%  **Critical –** ARDS, respiratory failure, shock, encephalopathy, myocardial injury or heart failure, coagulation dysfunction, AKI |
| Stordal, 2020^110^ | Norway | Database – mixed settings | <20 | 493 | Hospitalization; Death |  |
| Subcomisón de Epidemiología – Sociedad Argentina de Pediatria, 2020^111^ | Argentina | Database – mixed settings | <18 | 13,416 | ICU; Death |  |
| Surendra, 2021^112^ | Indonesia | Inpatients | <20 | 217 | Death |  |
| Tosca, 2021^113^ | Italy | Database – mixed settings | <18 | 198 | Hospitalization |  |
| Undurraga, 2021^114^ | Chile | Database – mixed settings | <20 | 40,418 | Death |  |
| Van der Zalm, 2020^115^ | South Africa | Hospital outpatients/inpatients | <14 | 159 | Hospitalization; ICU; Respiratory support; NIRS; IPPV; Death |  |
| Vergine, 2020^116^ | Italy | Hospital inpatients and community health houses | <19 | 194 | Hospitalization; ICU; Death |  |
| Whitworth, 2021^117^ | USA | Inpatients | <21 | 715 | ICU; IMV; ECMO; DVT; PE; Stroke; Major Bleed; Death |  |
| Yanover, 2020^118^ | Israel | Database – mixed settings | <18 | 647 | Severe Outcome; Death | ICU admission or death |
| Yilmaz, 2020^119^ | Turkey | Inpatients | <19 | 105 | ICU; Severe Outcome; Critical Outcome; Death | **Severe -** Fever and cough in the early period who develop dyspnea and central cyanosis within a week. **Critical –** ARDS, respiratory failure, shock, encephalopathy, myocardial involvement, coagulation defects, AKI |

ICU = intensive care unit; IMV = invasive mechanical ventilation; SpO_2.=_ oxygen saturation; ARDS = acute respiratory distress syndrome; ECMO = extracorporeal membrane oxygenation; ETT = endotracheal tube; BiPAP = bilevel positive pressure airway; MIS-C = multisystem inflammatory syndrome temporally associated with COVID-19; RRT = renal replacement therapy; AKI = acute kidney injury; GI = gastrointestinal; CXR = chest X-ray; CT = computed tomography; MI = myocardial infarction; CRP = C-reactive protein; ESR = erythrocyte sedimentation rate; P_a_O_2_ = partial pressure of oxygen; FiO_2_ = fraction of inspired oxygen; CPAP = continuous positive airway pressure; RDS = respiratory distress syndrome; DIC = disseminated intravascular coagulation; LOC = level of consciousness; PICU = pediatric intensive care unit; DVT = deep venous thrombosis; NIRS = near-infrared spectroscopy; IPPV = intermittent positive-pressure ventilation; PE = pulmonary embolism

**References**

1. Abayomi A, Abdus-Salam I, Adejumo M, et al. Presenting Symptoms and Predictors of Poor Outcomes Among 2,184 Patients with COVID-19 in Lagos State, Nigeria. *Int J Infect Dis*. 2021;102((Abayomi, Abdus-Salam, Adejumo, Agbolagorite, Lajide, Abdur-Razzaq) Lagos State Ministry of Health(Odukoya, Osibogun, Balogun, Oshodi) College of Medicine University of Lagos(Osibogun, Onasanya, Erinosho) Lagos State Primary Health Care Board(Osibogun, Os):226-232. doi:10.1016/j.ijid.2020.10.024

2. Afanasyeva OI, Kondratev VA, Dondurey EA, et al. Characteristics of CoVID-19 in children: The first experience in the hospital of st. Petersburg. *J Infektologii*. 2020;12(3):56-63. doi:10.22625/2072-6732-2020-12-3-56-63

3. Aguilera-Alonso D, Murias S, Martinez-De-Azagra Garde A, et al. Prevalence of thrombotic complications in children with SARS-CoV-2. *Arch Dis Child*. 2021;((Aguilera-Alonso) Pediatric Infectious Diseases Unit, Hospital General Universitario Gregorio Maranon, Madrid, Spain(Aguilera-Alonso) Instituto de Investigacion Sanitaria Gregorio Maranon (IiSGM), Hospital General Universitario Gregorio Maranon, Madrid, S). doi:10.1136/archdischild-2020-321351

4. Al Kuwari HM, Al Marri S, Al Romaihi HE, et al. Epidemiological investigation of the first 5685 cases of SARS-CoV-2 infection in Qatar, 28 February-18 April 2020. *BMJ Open*. 2020;10(10):e040428. doi:10.1136/bmjopen-2020-040428

5. Al Johani SM, Alharbi M, Kazzaz YM, et al. SARS-CoV-2 infection in children, clinical characteristics, diagnostic findings and therapeutic interventions at a tertiary care center in Riyadh, Saudi Arabia. *J Infect Public Health*. 2021;14(4):446-453. doi:10.1016/j.jiph.2020.12.034

6. Alonso GT, Ebekozien O, Gallagher MP, et al. Diabetic ketoacidosis drives COVID-19 related hospitalizations in children with type 1 diabetes. *J Diabetes*. Published online 2021. doi:10.1111/1753-0407.13184

7. Alsharrah D, Alhaddad F, Aljamaan S, et al. Clinical characteristics of pediatric SARS-CoV-2 infection and coronavirus disease 2019 (COVID-19) in Kuwait. *J Med Virol*. 2021;93(5):3246-3250. doi:10.1002/jmv.26684

8. Alswaidi FM, Assiri AM, Alhaqbani HH, Alalawi MM. Characteristics and outcome of COVID-19 cases in Saudi Arabia: Review of six-months of data (March-August 2020). *Saudi Pharm J*. 2021;((Alswaidi, Assiri, Alhaqbani, Alalawi) Assistant Agency for Preventive Health, MOH, Riyadh, Saudi Arabia). doi:10.1016/j.jsps.2021.04.030

9. Ansusinha E, Hahn A, Hamdy R, et al. Severe Coronavirus Disease-2019 in Children and Young Adults in the Washington, DC, Metropolitan Region. *J Pediatr*. 2020;223((DeBiasi, Ansusinha, Hahn, Hamdy, Harik, Hanisch, Jantausch, Koay) Division of Pediatric Infectious Diseases, Children’s National Hospital, Washington, DC, United States(DeBiasi, Song, Delaney, Bell, Smith, Pershad, Hahn, Hamdy, Harik, Hanisch, Jantausch,):199. doi:10.1016/j.jpeds.2020.05.007

10. Antunez-Montes OY, Escamilla MI, Figueroa-Uribe AF, et al. COVID-19 and Multisystem Inflammatory Syndrome in Latin American Children: A Multinational Study. *Pediatr Infect Dis J*. 2021;40(1):e1-e6. doi:10.1097/INF.0000000000002949

11. Armann JP, Doenhardt M, Berner R, et al. Hospital admission in children and adolescents with covid-19 early results from a national survey conducted by the German society for pediatric infectious diseases (DGPI). *Dtsch Arzteblatt Int*. 2020;117(21):373-374. doi:10.3238/arztebl.2020.0373

12. Arslan G, Akturk H, Duman M. Clinical Characteristics of Pediatric COVID-19 and Predictors of PCR Positivity. *Pediatr Int Off J Jpn Pediatr Soc*. 2021;((Arslan) Division of Pediatric Intensive Care, Department of Pediatrics, Derince Research and Training Hospital(Akturk) Division of Pediatric Infectious Disease, Department of Pediatrics, Derince Research and Training Hospital(Duman) Division of Pediatric). doi:10.1111/ped.14602

13. Atamari-Anahui N, Cruz-Nina ND, Condori-Huaraka M, et al. Characterization of coronavirus disease 2019 (COVID-19) in children and adolescents in Latin American and the Caribbean countries: A descriptive study. *Medwave*. 2020;20(8):e8025. doi:10.5867/medwave.2020.08.8025

14. Aykac K, Cura Yayla BC, Ozsurekci Y, et al. The association of viral load and disease severity in children with COVID-19. *J Med Virol*. 2021;93(5):3077-3083. doi:10.1002/jmv.26853

15. Aykac K, Cura Yayla BC, Ozsurekci Y, et al. Pneumococcal carriage in children with COVID-19. *Hum Vaccines Immunother*. 2021;17(6):1628-1634. doi:10.1080/21645515.2020.1849516

16. Bayesheva D, Boranbayeva R, Turdalina B, et al. COVID-19 in the paediatric population of Kazakhstan. *Paediatr Int Child Health*. 2021;41(1):76-82. doi:10.1080/20469047.2020.1857101

17. Bayramoglu E, Akkoc G, Agbas A, et al. The association between vitamin D levels and the clinical severity and inflammation markers in pediatric COVID-19 patients: single-center experience from a pandemic hospital. *Eur J Pediatr*. 2021;((Bayramoglu) Department of Pediatric Endocrinology, University of Health Sciences Haseki Training and Research Hospital, Istanbul, Turkey(Akkoc) Department of Pediatric Infectious Diseases, University of Health Sciences Haseki Training and Research Hospit). doi:10.1007/s00431-021-04030-1

18. Bellino S, Rota MC, Riccardo F, et al. Pediatric COVID-19 cases prelockdown and postlockdown in Italy. *Pediatrics*. 2021;147(2):e2020035238. doi:10.1542/peds.2020-035238

19. Blot M, Piroth L, Cottenet J, et al. Comparison of the characteristics, morbidity, and mortality of COVID-19 and seasonal influenza: a nationwide, population-based retrospective cohort study. *Lancet Respir Med*. 2021;9(3):251-259. doi:10.1016/S2213-2600%2820%2930527-0

20. Brenner EJ, Kappelman MD, Zhang X, et al. Benign Evolution of SARS-Cov2 Infections in Children With Inflammatory Bowel Disease: Results From Two International Databases. *Clin Gastroenterol Hepatol*. 2021;19(2):394. doi:10.1016/j.cgh.2020.10.010

21. Bruno C, Camacho PEF, Bailey LC, et al. Assessment of 135794 Pediatric Patients Tested for Severe Acute Respiratory Syndrome Coronavirus 2 across the United States. *JAMA Pediatr*. 2021;175(2):176-184. doi:10.1001/jamapediatrics.2020.5052

22. Calvo C, Udaondo C. Covid-19 in Children with Rheumatic Diseases in the Spanish National Cohort EPICO-AEP. *J Rheumatol*. 2021;((Calvo) Pediatric and Infectious Disease Unit. Hospital Universtiario La Paz and La Paz Research Institute (IdiPaz). Madrid, Spain. Traslational Research Network of Pediatric Infectious Diseases (RITIP). Madrid. Spain. Pediatric Rheumatology Department, H). doi:10.3899/jrheum.201548

23. Camara E, Diallo FB, Diop MM, et al. Epidemiological and clinical profile of children with coronavirus disease (Covid-19) at the center for the treatment of epidemics and infection prevention (cteip) of the university hospital of donka in conakry. *Pan Afr Med J*. 2020;37((Camara, Diallo, Diop, Cherif, Kouyate, Bangoura, Barry, Ngadande, Kaba, Kolie, Camara, Diallo) Service de Pediatrie, Hopital National Donka, Conakry, Equatorial Guinea(Barry, Diallo) Institut de Nutrition et de Sante de l’Enfant, Conakry, Equatorial Guin):363. doi:10.11604/PAMJ.2020.37.363.26211

24. Castro MC, Gurzenda S, Macario EM, Franca GVA. Characteristics, outcomes and risk factors for mortality of 522 167 patients hospitalised with COVID-19 in Brazil: A retrospective cohort study. *BMJ Open*. 2021;11(5):e049089. doi:10.1136/bmjopen-2021-049089

25. Chua GT, Rosa Duque JS, Wong WHS, et al. Clinical Characteristics and Transmission of COVID-19 in Children and Youths during 3 Waves of Outbreaks in Hong Kong. *JAMA Netw Open*. 2021;((Chua, Rosa Duque, Wong, Ho, Tso, Tung, Leung, Chan, Chow, Lau, Ip) Department of Paediatrics and Adolescent Medicine, Li Ka Shing Faculty of Medicine, University of Hong Kong, Queen Mary Hospital, 102 Pokfulam Rd, Pokfulam, Hong Kong(Wong, Lam, Kwan) Pae). doi:10.1001/jamanetworkopen.2021.8824

26. Cofre F, Mackenney J, Poli C, et al. Clinical manifestations of SARS-CoV-2 infection in children in the middle of pandemic season in a pediatric tertiary center. Report of local COVID Clinical Committee, Hospital de Ninos Roberto del Rio, Santiago Chile. *Rev Chil Infectologia Organo Of Soc Chil Infectologia*. 2020;37(6):756-761. doi:10.4067/S0716-10182020000600756

27. COVID-19 Australia: Epidemiology Report 30: Fortnightly reporting period ending 22 November 2020. *Commun Dis Intell 2018*. 2020;44. doi:10.33321/cdi.2020.44.91

28. De la Hoz-Restrepo F, Alvis-Zakzuk NJ, De la Hoz-Gomez JF, De la Hoz A, Alvis-Guzman N, Gomez Del Corral L. Is Colombia an example of successful containment of the 2020 COVID-19 pandemic? A critical analysis of the epidemiological data, March to July 2020. *Int J Infect Dis*. 2020;99((De la Hoz-Restrepo) Department of Public Health, Universidad Nacional de Colombia, Carrera 10 No. 93-51 apto 505, Bogota, DC, Colombia(Alvis-Zakzuk) Department of Economic Sciences, Universidad de la Costa-CUC, Calle 58 No. 55-66, Barranquilla, Colombi):522-529. doi:10.1016/j.ijid.2020.08.017

29. Deng G, Yin M, Chen X, Zeng F. Clinical determinants for fatality of 44,672 patients with COVID-19. *Crit Care*. 2020;24(1):179. doi:10.1186/s13054-020-02902-w

30. Di Fusco M, Shea KM, Nguyen JL, et al. Health outcomes and economic burden of hospitalized COVID-19 patients in the United States. *J Med Econ*. 2021;24(1):308-317. doi:10.1080/13696998.2021.1886109

31. Ece I, Kocoglu M, Kavurt AV, et al. Assessment of Cardiac Arrhythmic Risk in Children With Covid-19 Infection. *Pediatr Cardiol*. 2021;42(2):264-268. doi:10.1007/s00246-020-02474-0

32. Elimian KO, Ochu CL, Ilori E, et al. Descriptive epidemiology of coronavirus disease 2019 in Nigeria, 27 February-6 June 2020. *Epidemiol Infect*. 2020;148:e208. doi:10.1017/s095026882000206x

33. Finelli L, Gupta V, Petigara T, Yu K, Bauer KA, Puzniak LA. Mortality Among US Patients Hospitalized With SARS-CoV-2 Infection in 2020. *JAMA Netw Open*. 2021;4(4):e216556. doi:10.1001/jamanetworkopen.2021.6556

34. Floyd GC, Dudley JW, Xiao R, et al. Prevalence of asthma in hospitalized and non-hospitalized children with COVID-19. *J Allergy Clin Immunol Pract*. 2021;((Floyd, Feudtner, Kenyon) PolicyLab and Center for Pediatric Clinical Effectiveness, Children’s Hospital of Philadelphia, Philadelphia, Pa, United States(Dudley) Department of Biomedical and Health Informatics, Children’s Hospital of Philadelphia, Philade). doi:10.1016/j.jaip.2021.02.038

35. Freeman MC, Gaietto K, DiCicco LA, et al. A Comprehensive Clinical Description of Pediatric SARS-CoV-2 Infection in Western Pennsylvania. *medRxiv*. Published online 2020. doi:10.1101/2020.12.14.20248192

36. Gaborieau L, Delestrain C, Epaud R, et al. Epidemiology and clinical presentation of children hospitalized with SARS-CoV-2 infection in suburbs of paris. *J Clin Med*. 2020;9(7):1-10. doi:10.3390/jcm9072227

37. Garazzino S, Denina M, Tovo PA, et al. Epidemiology, Clinical Features and Prognostic Factors of Pediatric SARS-CoV-2 Infection: Results From an Italian Multicenter Study. *Front Pediatr*. 2021;9((Garazzino, Denina, Tovo, Pruccoli) Pediatric Infectious Diseases Unit, Regina Margherita Children’s Hospital, University of Turin, Turin, Italy(Lo Vecchio, Pierri) Section of Pediatrics, Department of Translational Medical Science, University of Naples F):649358. doi:10.3389/fped.2021.649358

38. Geng MJ, Wang LP, Ren X, et al. Risk factors for developing severe COVID-19 in China: an analysis of disease surveillance data. *Infect Dis Poverty*. 2021;10(1):48. doi:10.1186/s40249-021-00820-9

39. Giacomet V, Barcellini L, Stracuzzi M, et al. Gastrointestinal symptoms in severe covid-19 children. *Pediatr Infect Dis J*. 2020;((Giacomet, Barcellini, Stracuzzi) Paediatric Infectious Disease Unit, Department of Pediatrics, Luigi Sacco Hospital, University of Milan, Milan, Italy(Longoni, Folgori) Department of Pediatrics, V. Buzzi Children’s Hospital, University of Milan, Milan, I):E317-E320. doi:10.1097/INF.0000000000002843

40. Gottlieb M, Ward E, Sansom S, Hota B, Frankenberger C. Clinical Course and Factors Associated With Hospitalization and Critical Illness Among COVID-19 Patients in Chicago, Illinois. *Acad Emerg Med*. 2020;27(10):963-973. doi:10.1111/acem.14104

41. Gotzinger F, Bogyi M, Lanaspa M, et al. COVID-19 in children and adolescents in Europe: a multinational, multicentre cohort study. *Lancet Child Adolesc Health*. 2020;4(9):653-661. doi:10.1016/S2352-4642%2820%2930177-2

42. Graff K, Smith C, Silveira L, et al. Risk Factors for Severe COVID-19 in Children. *Pediatr Infect Dis J*. 2021;((Graff, Smith, Silveira, Curran-Hays, Jarjour, Mattiucci, McFarland, Dominguez, Abuogi) Department of Pediatrics, School of Medicine, University of Colorado, Aurora, CO, United States(Jung, Dominguez) Department of Pathology and Laboratory Medicine, Child):E137-E145. doi:10.1097/INF.0000000000003043

43. Green MS, Peer V, Schwartz N, Nitzan D. The confounded crude case-fatality rates (CFR) for COVID-19 hide more than they reveal-a comparison of age-specific and age-adjusted CFRs between seven countries. *PLoS One*. 2020;15(10):e0241031. doi:10.1371/journal.pone.0241031

44. Green MS, Nitzan D, Schwartz N, Niv Y, Peer V. Sex differences in the case-fatality rates for COVID-19-A comparison of the age-related differences and consistency over seven countries. *PLoS One*. 2021;16(4):e0250523. doi:10.1371/journal.pone.0250523

45. Guo CX, Yang GP, He L, et al. Epidemiological and clinical features of pediatric COVID-19. *BMC Med*. 2020;18(1):250. doi:10.1186/s12916-020-01719-2

46. Guo L, Zhang J, Liao C, et al. Clinical analysis and pluripotent stem cells-based model reveal possible impacts of ACE2 and lung progenitor cells on infants vulnerable to COVID-19. *Theranostics*. 2021;11(5):2170-2181. doi:10.7150/thno.53136

47. Guo L, Li Z, Zhou X, et al. Distinct disease severity between children and older adults with COVID-19: Impacts of ACE2 expression, distribution, and lung progenitor cells. *Clin Infect Dis Off Publ Infect Dis Soc Am*. 2021;((Zhang, Guo, Huang, Li, Zhou, Zhang, Liu, Yang, Wu, Zhang, Liao, Lian) Prenatal Diagnostic Centre and Cord Blood Bank; Guangzhou Women and Children’s Medical Center, Guangzhou Medical University, Guangzhou, China(Zhang, Yan Ma, Tse, Lian) Department of Me). doi:10.1093/cid/ciaa1911

48. Hammadi S, AlKanan AK, Fares M, et al. Basrah Preliminary Experience With COVID-19: A Report on 6404 Patients. *Cureus*. 2021;13(1):e13012. doi:10.7759/cureus.13012

49. Haw NJL, Uy J, Abrigo MRM, Sy KTL. Epidemiological profile and transmission dynamics of COVID-19 in the philippines. *Epidemiol Infect*. 2020;((Haw, Uy) Health Sciences Program, School of Science and Engineering, Ateneo de Manila University, Katipunan Avenue, Loyola Heights, Quezon City 1108, Philippines(Uy, Abrigo) Philippine Institute for Development Studies, Philippines(Sy) Department of Epid). doi:10.1017/S0950268820002137

50. Hernandez-Garduno E. Comorbidities that predict acute respiratory syndrome coronavirus 2 test positivity in Mexican Children: A case-control study. *Pediatr Obes*. 2021;16(5):e12740. doi:10.1111/ijpo.12740

51. Heston SM, Aquino JN, Carr ST, et al. SARS-CoV-2 Infections Among Children in the Biospecimens from Respiratory Virus-Exposed Kids (BRAVE Kids) Study. *Clin Infect Dis Off Publ Infect Dis Soc Am*. 2020;((Hurst, Heston, Aquino, Carr, Jenkins, Pfeiffer, Cunningham, Steinbach, Lugo, Moody, Permar, Kelly) Department of Pediatrics, Division of Infectious Diseases, Duke University School of Medicine, Durham(Hurst, Crew, Permar) Children’s Health and Discovery). doi:10.1093/cid/ciaa1693

52. Heudorf U, Steul K, Gottschalk R. Sars-Cov-2 in children - insights and conclusions from the mandatory reporting data in Frankfurt am Main, Germany, March-July 2020. *GMS Hyg Infect Control*. 2020;15:Doc24. doi:10.3205/dgkh000359

53. Hijazi LO, Alaraifi AK, Alsaab F. Otolaryngology manifestations of COVID-19 in pediatric patients. *Int J Pediatr Otorhinolaryngol*. 2021;144((Hijazi, Alaraifi, Alsaab) Division of Otolaryngology-Head and Neck Surgery, Department of Surgery, Ministry of National Guard Health Affairs, King Abdullah International Medical Research Center, Riyadh, Saudi Arabia):110701. doi:10.1016/j.ijporl.2021.110701

54. Hon KL, Leung KK. Paediatrics is a big player of covid-19 in Hong Kong. *Hong Kong Med J*. 2020;26(3):265-266. doi:10.12809/hkmj208546

55. Howard LM, Garguilo K, Gillon J, et al. Characteristics and clinical features of SARS-CoV-2 infections among ambulatory and hospitalized children and adolescents in an integrated health care system in Tennessee. *medRxiv*. Published online 2020. doi:10.1101/2020.10.08.20208751

56. Jefferies S, Gilkison C, Graham G, et al. COVID-19 in New Zealand and the impact of the national response: a descriptive epidemiological study. *Lancet Public Health*. 2020;5(11):e612-e623. doi:10.1016/S2468-2667%2820%2930225-5

57. Jeong GH, Lee HJ, Yoon S, et al. Effective control of COVID-19 in South Korea: Cross-sectional study of epidemiological data. *J Med Internet Res*. 2020;22(12):e22103. doi:10.2196/22103

58. Kamdar KY, Kim TO, Doherty EE, et al. COVID-19 outcomes in a large pediatric hematology-oncology center in Houston, Texas. *Pediatr Hematol Oncol*. Published online 2021:1-14. doi:10.1080/08880018.2021.1924327

59. Krajcar N, Stemberger Marić L, Šurina A, et al. Epidemiological and clinical features of Croatian children and adolescents with a PCR-confirmed coronavirus disease 2019: differences between the first and second epidemic wave. *Croat Med J*. 2020;61(6):491-500. doi:10.3325/cmj.2020.61.491

60. Krishnasamy N, Natarajan M, Ramachandran A, et al. Clinical Outcomes among Asymptomatic or Mildly Symptomatic COVID-19 Patients in an Isolation Facility in Chennai, India. *Am J Trop Med Hyg*. 2021;104(1):85-90. doi:10.4269/ajtmh.20-1096

61. Kushner LE, Schroeder AR, Kim J, Mathew R. “For COVID” or “With COVID”: Classification of SARS-CoV-2 Hospitalizations in Children. *Hosp Pediatr*. Published online 2021. doi:10.1542/hpeds.2021-006001

62. Laxminarayan R, Wahl B, Dudala SR, et al. Epidemiology and transmission dynamics of COVID-19 in two Indian states. *Science*. 2020;370(6517):691-697. doi:10.1126/science.abd7672

63. Lazzerini M, Lega S, Conte M, et al. Characteristics and risk factors for SARS-CoV-2 in children tested in the early phase of the pandemic: A cross-sectional study, Italy, 23 February to 24 May 2020. *Eurosurveillance*. 2021;26(14):1-12. doi:10.2807/1560-7917.ES.2021.26.14.2001248

64. Leeb RT, Price S, Sliwa S, et al. COVID-19 Trends Among School-Aged Children - United States, March 1-September 19, 2020. *MMWR Morb Mortal Wkly Rep*. 2020;69(39):1410-1415. doi:10.15585/mmwr.mm6939e2

65. Leidman E, Duca LM, Omura JD, Proia K, Stephens JW, Sauber-Schatz EK. COVID-19 Trends Among Persons Aged 0-24 Years - United States, March 1-December 12, 2020. *MMWR Morb Mortal Wkly Rep*. 2021;70(3):88-94. doi:10.15585/mmwr.mm7003e1

66. Liu X, Li W, Chen J, et al. Comparative study of hospitalized children with acute respiratory distress syndrome caused by SARS-CoV-2 and influenza virus. *BMC Infect Dis*. 2021;21(1):412. doi:10.1186/s12879-021-06068-w

67. Lu X, Zhang L, Du H, et al. SARS-CoV-2 infection in children. *N Engl J Med*. 2020;382(17):1663-1665. doi:10.1056/NEJMc2005073

68. Malagon-Rojas J, Alvarez S, Ibanez E, Parra ELB, Toloza-Perez YG, Mercado M. Analysis of COVID-19 mortality and survival in Colombia: A prospective cohort study. *Infectio*. 2021;25(3):176-181. doi:10.22354/IN.V25I3.943

69. Maltezou HC, Magaziotou I, Dedoukou X, et al. Children and Adolescents with SARS-CoV-2 Infection: Epidemiology, Clinical Course and Viral Loads. *Pediatr Infect Dis J*. 2020;((Maltezou) Directorate for Research, Studies, and Documentation, National Public Health Organization, 3-5 Agrafon Street, Athens 15123, Greece(Magaziotou, Dedoukou) Directorate of Epidemiological Surveillance and Interventions for Infectious Diseases, Nat):E388-E392. doi:10.1097/INF.0000000000002899

70. Mangia C, Russo A, Civitelli S, Gianicolo EAL. Sex/gender differences in COVID-19 lethality: what the data say, and do not say. *Epidemiol Prev*. 2020;44(56 Supplement 2):400-406. doi:10.19191/EP20.5-6.S2.145

71. Marcello RK, Dolle J, Grami S, et al. Characteristics and outcomes of COVID-19 patients in New York City’s public hospital system. *PLoS ONE*. 2020;15(12 December):e0243027. doi:10.1371/journal.pone.0243027

72. Martins-Filho PR, Quintans-Junior LJ, de Souza Araujo AA, et al. Socio-economic inequalities and COVID-19 incidence and mortality in Brazilian children: a nationwide register-based study. *Public Health*. 2021;190((Martins-Filho, Quintans-Junior, de Souza Araujo, Sposato, Souza Tavares, Gurgel, Fontes Leite) Federal University of Sergipe, Brazil(Sposato) Juvenile Justice and Child Abuse, UNICEF, Brazil(de Paiva) Federal University of Minas Gerais, Brazil(Santos) Un):4-6. doi:10.1016/j.puhe.2020.11.005

73. McPherson ML, Krennerich EC, Arrington AS, Graf JM, Sitler SG. Safe Ground Transport of Pediatric COVID-19 Patients-A Single-Center First-Surge Experience. *Pediatr Emerg Care*. 2021;37(3):175-178. doi:10.1097/PEC.0000000000002330

74. Merzon E, Vinker S, Golan Cohen A, et al. The Association between ADHD and the Severity of COVID-19 Infection. *J Atten Disord*. 2021;((Merzon, Vinker, Golan Cohen, Green) Tel-Aviv, Israel(Merzon, Vinker, Golan Cohen, Green, Weizman, Manor) Tel Aviv University, Israel(Weiss) Cambridge Health Alliance, MA, United States(Cortese) Center for Innovation in Mental Health, School of Psychology):10870547211003660. doi:10.1177/10870547211003659

75. Moeller A, Thanikkel L, Pijnenburg MWH, et al. COVID-19 in children with underlying chronic respiratory diseases: Survey results from 174 centres. *ERJ Open Res*. 2020;6(4):1-8. doi:10.1183/23120541.00409-2020

76. More K, Chawla D, Murki S, Tandur B, Deorari AK, Kumar P. Outcomes of Neonates Born to Mothers with Coronavirus Disease 2019 (COVID-19) - National Neonatology Forum (NNF) India COVID-19 Registry. *Indian Pediatr*. 2021;((More) Sidra Medicine Hospital, Doha, Qatar(Chawla) Government Medical College, Chandigarh, India(Murki) Paramitha Children Hospital, Hyderabad, Telangana, India(Tandur) Princess Durru Shehvar Children’s and General Hospital, India(Deorari) All India Inst).

77. Murk W, Gierada M, Weckstein A, Rassen JA, Fralick M, Klesh R. Diagnosis-wide analysis of COVID-19 complications: an exposure-crossover study. *CMAJ*. 2021;193(1):E10-E18. doi:10.1503/cmaj.201686

78. Nikolaeva SV, Akimkin VG, Gorelov AV. New coronavirus infection covid-19: Features of the course in children in the russian federation. *Pediatr - Zhurnal Im GN Speranskogo*. 2020;99(6):57-62. doi:10.24110/0031-403X-2020-99-6-57-62

79. Oh B, Hwangbo S, Jung T, et al. Prediction Models for the Clinical Severity of Patients With COVID-19 in Korea: Retrospective Multicenter Cohort Study. *J Med Internet Res*. 2021;23(4):e25852. doi:10.2196/25852

80. Omrani AS, Almaslamani MA, Daghfal J, et al. The first consecutive 5000 patients with Coronavirus Disease 2019 from Qatar; a nation-wide cohort study. *BMC Infect Dis*. 2020;20(1):777. doi:10.1186/s12879-020-05511-8

81. Otiken Arikan K, Sahinkaya S, Boncuoglu E, et al. Can Hematological Findings of COVID-19 in Pediatric Patients Guide Physicians about Clinical Severity? *Turk J Haematol Off J Turk Soc Haematol*. 2021;((Otiken Arikan, Sahinkaya, Boncuoglu, Kiymet, Cem, Akaslan Kara, Bayram, Devrim) University of Health Sciences Turkey, Dr. Behcet Uz Children Diseases and Surgery Training and Research Hospital, Clinic of Pediatric Infectious Diseases, Izmir, Turkey). doi:10.4274/tjh.galenos.2021.2021.0157

82. Otto WR, Posch LC, Geoghegan S, et al. The epidemiology of severe acute respiratory syndrome coronavirus 2 in a pediatric healthcare network in the United States. *J Pediatr Infect Dis Soc*. 2020;9(5):523-529. doi:10.1093/JPIDS/PIAA074

83. Owusu M, Sylverken AA, Ankrah ST, et al. Epidemiological profile of SARS-CoV-2 among selected regions in Ghana: A cross-sectional retrospective study. *PLoS One*. 2020;15(12):e0243711. doi:10.1371/journal.pone.0243711

84. Guner Ozenen G, Sahbudak Bal Z, Umit Z, et al. Demographic, clinical, and laboratory features of COVID-19 in children: The role of mean platelet volume in predicting hospitalization and severity. *J Med Virol*. 2021;93(5):3227-3237. doi:10.1002/jmv.26902

85. Paquette D, Bell C, Roy M, et al. Laboratory-confirmed COVID-19 in children and youth in Canada, January 15-April 27, 2020. *Can Commun Rep*. 2020;46(5):121-124. doi:10.14745/ccdr.v46i06a04

86. Parcha V, Arora G, Booker KS, et al. A retrospective cohort study of 12,306 pediatric COVID-19 patients in the United States. *Sci Rep*. 2021;11(1):10231. doi:10.1038/s41598-021-89553-1

87. Parri N, Lenge M, Cantoni B, et al. COVID-19 in 17 Italian Pediatric Emergency Departments. *Pediatrics*. 2020;146(6). doi:10.1542/peds.2020-1235

88. Parri N, Masi S, Magista AM, et al. Characteristic of COVID-19 infection in pediatric patients: early findings from two Italian Pediatric Research Networks. *Eur J Pediatr*. 2020;179(8):1315-1323. doi:10.1007/s00431-020-03683-8

89. Pereda R, Gonzalez D, Rivero HB, et al. Therapeutic Effectiveness of Interferon Alpha 2b Treatment for COVID-19 Patient Recovery. *J Interferon Cytokine Res*. 2020;40(12):578-588. doi:10.1089/jir.2020.0188

90. Picão de Carvalho C, Castro C, Sampaio Graça I, et al. [Case Series of 103 Children with SARS-CoV-2 Infection in Portugal]. *Acta Med Port*. 2020;33(12):795-802. doi:10.20344/amp.14537

91. Pinninti SG, Pati S, Poole C, et al. Virological characteristics of hospitalized children with SARS-CoV-2 infection. *Pediatrics*. 2021;147(5):e2020037812. doi:10.1542/peds.2020-037812

92. Powell AA, Amin-Chowdhury Z, Mensah A, Saliba V, Ramsay ME, Ladhani SN. Severe Acute Respiratory Syndrome Coronavirus 2 Infections in Primary School Age Children after Partial Reopening of Schools in England. *Pediatr Infect Dis J*. 2021;((Powell, Amin-Chowdhury, Mensah, Ramsay, Saliba, Ladhani) Division of Immunisation and Countermeasures, Public Health England, 61 Colindale Avenue, London NW9 5EQ, United Kingdom(Ramsay) London School of Hygiene and Tropical Medicine, United Kingdom(Ladha):E243-E245. doi:10.1097/INF.0000000000003120

93. Preston LE, Chevinsky JR, Kompaniyets L, et al. Characteristics and Disease Severity of US Children and Adolescents Diagnosed With COVID-19. *JAMA Netw Open*. 2021;4(4):e215298. doi:10.1001/jamanetworkopen.2021.5298

94. Priya S, Selva Meena M, Brinda Priyadharshini C, Vijay Anand V, Sangumani J, Rathinam P. Factors influencing the outcome of COVID-19 patients admitted in a tertiary care hospital, Madurai.- a cross-sectional study. *Clin Epidemiol Glob Health*. 2021;10((Priya, Selva Meena, Brinda Priyadharshini, Vijay Anand) Institute of Community Medicine, Madurai Medical College, India(Sangumani) Government RajajiHospital(GRH) and Madurai Medical College, India(Rathinam) COVID19 & HOD of the Department of Respiratory):100705. doi:10.1016/j.cegh.2021.100705

95. Rabha AC, Oliveira Junior FI, Oliveira TA, et al. CLINICAL MANIFESTATIONS OF CHILDREN AND ADOLESCENTS WITH COVID-19: REPORT OF THE FIRST 115 CASES FROM SABARÁ HOSPITAL INFANTIL. *Rev Paul Pediatr*. 2020;39:e2020305. doi:10.1590/1984-0462/2021/39/2020305

96. Raciborski F, Pinkas J, Jankowski M, et al. Dynamics of the coronavirus disease 2019 outbreak in Poland: an epidemiological analysis of the first 2 months of the epidemic. *Pol Arch Intern Med*. 2020;130(7-8):615-621. doi:10.20452/pamw.15430

97. Rao S, Gavali V, Prabhu SS, et al. Outcome of Children Admitted With SARS-CoV-2 Infection: Experiences From a Pediatric Public Hospital. *Indian Pediatr*. 2021;58(4):358-362. doi:10.1007/s13312-021-2196-4

98. Redondo-Bravo L, Moros MJS, Sanchez EVM, et al. The first wave of the COVID-19 pandemic in Spain: Characterisation of cases and risk factors for severe outcomes, as at 27 April 2020. *Eurosurveillance*. 2020;25(50):e2001431. doi:10.2807/1560-7917.ES.2020.25.50.2001431

99. Reilev M, Kristensen KB, Lund LC, et al. Characteristics and predictors of hospitalization and death in the first 11 122 cases with a positive RT-PCR test for SARS-CoV-2 in Denmark: A nationwide cohort. *Int J Epidemiol*. 2020;49(5):1468-1481. doi:10.1093/ije/dyaa140

100. Saleh NY, Aboelghar HM, Salem SS, et al. The severity and atypical presentations of COVID-19 infection in pediatrics. *BMC Pediatr*. 2021;21(1):144. doi:10.1186/s12887-021-02614-2

101. Saraiva BM, Garcia AM, Silva TM, Gouveia C, Brito MJ. Clinical and therapeutic approach to hospitalized COVID-19 patients: A pediatric cohort in Portugal. *Acta Med Port*. 2021;34(4):283-290. doi:10.20344/AMP.15360

102. Semenova Y, Glushkova N, Pivina L, et al. Epidemiological Characteristics and Forecast of COVID-19 Outbreak in the Republic of Kazakhstan. *J Korean Med Sci*. 2020;35(24):e227. doi:10.3346/jkms.2020.35.e227

103. Sharif E, Narchi H, Elghoudi A, Aldhanhani H, Ghatasheh G. Covid-19 in Children and Young Adolescents in Al Ain, United Arab Emirates- a Retrospective Cross-Sectional Study. *Front Pediatr*. 2020;8((Elghoudi, Sharif) Department of Pediatrics, Al Ain Hospital, Al Ain, United Arab Emirates(Elghoudi, Sharif, Narchi) The Department of Paediatrics, College of Medicine and Health Sciences, UAE University, Al Ain, United Arab Emirates(Elghoudi) Department):603741. doi:10.3389/fped.2020.603741

104. Sharma AK, Chapagain RH, Bista KP, et al. Epidemiological and clinical profile of covid-19 in nepali children: An initial experience. *J Nepal Paediatr Soc*. 2020;40(3):202-209. doi:10.3126/jnps.v40i3.32438

105. Shim E. Regional variability in covid-19 case fatality rate in canada, february-december 2020. *Int J Environ Res Public Health*. 2021;18(4):1-10. doi:10.3390/ijerph18041839

106. Siddiqui M, Gültekingil A, Bakırcı O, Uslu N, Baskın E. Comparison of clinical features and laboratory findings of coronavirus disease 2019 and influenza A and B infections in children: a single-center study. *Clin Exp Pediatr*. Published online 2021. doi:10.3345/cep.2021.00066

107. Silverii GA, Monami M, Cernigliaro A, et al. Are diabetes and its medications risk factors for the development of COVID-19? Data from a population-based study in Sicily. *Nutr Metab Cardiovasc Dis*. 2021;31(2):396-398. doi:10.1016/j.numecd.2020.09.028

108. Soriano-Arandes A, Soler-Palacin P, Gatell A, et al. Household SARS-CoV-2 transmission and children: a network prospective study. *Clin Infect Dis Off Publ Infect Dis Soc Am*. 2021;((Soriano-Arandes, Soler-Palacin) Pediatric Infectious Diseases and Immunodeficiencies Unit, Hospital Universitari Vall d’Hebron, Barcelona, Spain(Gatell, Serrano, Valldeperez) Equip Pediatria Territorial Alt Penedes-Garraf, Barcelona, Spain(Biosca, Capdev). doi:10.1093/cid/ciab228

109. Soysal A, Gönüllü E, Arslan H, et al. Comparison of clinical and laboratory features and treatment options of 237 Comparison of clinical and laboratory features and treatment options of 237 symptomatic and asymptomatic children infected with SARS-CoV-2 in the early phase of the COVID-19 pandemic in Turkey. *Jpn J Infect Dis*. Published online 2020. doi:10.7883/yoken.JJID.2020.781

110. Stordal K, Bakken IJ, Greve-Isdahl M, et al. SARS-CoV-2 in children and adolescents in Norway: confirmed infection, hospitalisations and underlying conditions. *Tidsskr Den Nor Laegeforening Tidsskr Prakt Med Ny Raekke*. 2020;140(11). doi:10.4045/tidsskr.20.0457

111. Anonymous. Epidemiological update of COVID-19 in Pediatrics. Epidemiological week 32. *Arch Argent Pediatr*. 2020;118(4bis):C16-C22.

112. Surendra H, Elyazar IR, Djaafara BA, et al. Clinical characteristics and mortality associated with COVID-19 in Jakarta, Indonesia: A hospital-based retrospective cohort study. *Lancet Reg Health West Pac*. 2021;9:100108. doi:10.1016/j.lanwpc.2021.100108

113. Tosca MA, Licari A, Marseglia GL, Ciprandi G. COVID-19 in Italian children and adolescents: The role of allergy and asthma. *Allergy Asthma Proc*. 2021;42(3):e101-e102. doi:10.2500/aap.2021.42.210023

114. Undurraga EA, Chowell G, Mizumoto K. COVID-19 case fatality risk by age and gender in a high testing setting in Latin America: Chile, March-August 2020. *Infect Dis Poverty*. 2021;10(1):11. doi:10.1186/s40249-020-00785-1

115. van der Zalm MM, Workman JJ, Lishman J, et al. Clinical experience with SARS CoV-2 related illness in children - hospital experience in Cape Town, South Africa. *Clin Infect Dis Off Publ Infect Dis Soc Am*. 2020;((van der Zalm, Workman) Desmond Tutu TB Centre, Department of Paediatrics and Child Health, Faculty of Medicine and Health Sciences, Stellenbosch University, South Africa(Lishman, Verhagen, Redfern, Smit, Barday, Ruttens, da Costa, van Jaarsveld, Itana, P). doi:10.1093/cid/ciaa1666

116. Vergine G, Fantini M, Sambri V, et al. Home management of children with COVID-19 in the Emilia-Romagna region, Italy. *Front Pediatr*. 2020;8((Vergine) Department of Pediatrics, Infermi Hospital Rimini, Azienda Sanitaria Locale Romagna, Rimini, Italy(Fantini, Sambri) Unit of Microbiology, The Great Romagna Area Hub Laboratory, Pievesestina di Cesena, Italy(Marchetti) Department of Pediatrics, S):1-5. doi:10.3389/fped.2020.575290

117. Whitworth HB, Ballester L, Diorio C, et al. Rate of thrombosis in children and adolescents hospitalized with COVID-19 or MIS-C. *Blood*. 2021;((Whitworth, Ballester, Diorio, Raffini) Children’s Hospital of Philadelphia, Philadelphia, PA, United States(Sartain, Cohen) Baylor College of Medicine, Houston, TX, United States(Kumar, Randolph) Boston Children’s Hospital, Boston, MA, United States(Arms). doi:10.1182/blood.2020010218

118. Yanover C, Mizrahi B, Kalkstein N, et al. What Factors Increase the Risk of Complications in SARS-CoV-2-Infected Patients? A Cohort Study in a Nationwide Israeli Health Organization. *JMIR Public Health Surveill*. 2020;6(3):e20872. doi:10.2196/20872

119. Yilmaz K, Gozupirinccioglu A, Aktar F, et al. Evaluation of the novel coronavirus disease in Turkish children: Preliminary outcomes. *Pediatr Pulmonol*. 2020;55(12):3587-3594. doi:10.1002/ppul.25095
